# BrainFreeze: Expanding the Capabilities of Neuromorphic Systems Using Mixed-Signal Superconducting Electronics

**DOI:** 10.3389/fnins.2021.750748

**Published:** 2021-12-21

**Authors:** Paul Tschirhart, Ken Segall

**Affiliations:** ^1^Advanced Technology Laboratory, Northrop Grumman, Linthicum, MD, United States; ^2^Department of Physics and Astronomy, Colgate University, Hamilton, NY, United States

**Keywords:** neuromorphic, architecture, superconducting, mixed-signal, spiking

## Abstract

Superconducting electronics (SCE) is uniquely suited to implement neuromorphic systems. As a result, SCE has the potential to enable a new generation of neuromorphic architectures that can simultaneously provide scalability, programmability, biological fidelity, on-line learning support, efficiency and speed. Supporting all of these capabilities simultaneously has thus far proven to be difficult using existing semiconductor technologies. However, as the fields of computational neuroscience and artificial intelligence (AI) continue to advance, the need for architectures that can provide combinations of these capabilities will grow. In this paper, we will explain how superconducting electronics could be used to address this need by combining analog and digital SCE circuits to build large scale neuromorphic systems. In particular, we will show through detailed analysis that the available SCE technology is suitable for near term neuromorphic demonstrations. Furthermore, this analysis will establish that neuromorphic architectures built using SCE will have the potential to be significantly faster and more efficient than current approaches, all while supporting capabilities such as biologically suggestive neuron models and on-line learning. In the future, SCE-based neuromorphic systems could serve as experimental platforms supporting investigations that are not feasible with current approaches. Ultimately, these systems and the experiments that they support would enable the advancement of neuroscience and the development of more sophisticated AI.

## 1. Introduction

Superconducting electronics (SCE) has many characteristics that make it a natural fit for implementing neuromorphic systems. This work will explore how such a system might be constructed using a combination of digital and analog SCE. The unique collection of capabilities enabled by SCE neuromorphic systems has the potential to provide solutions to some of the most difficult problems facing AI research in the future. In particular, the efficiency and speed of SCE architectures could help to address the compute challenges facing AI development. Similarly, the biological fidelity that is possible in SCE-based neural circuits may also prove to be a valuable source of future capabilities as researchers seek to incorporate novel functionality into their neural networks.

**Figure 1:**
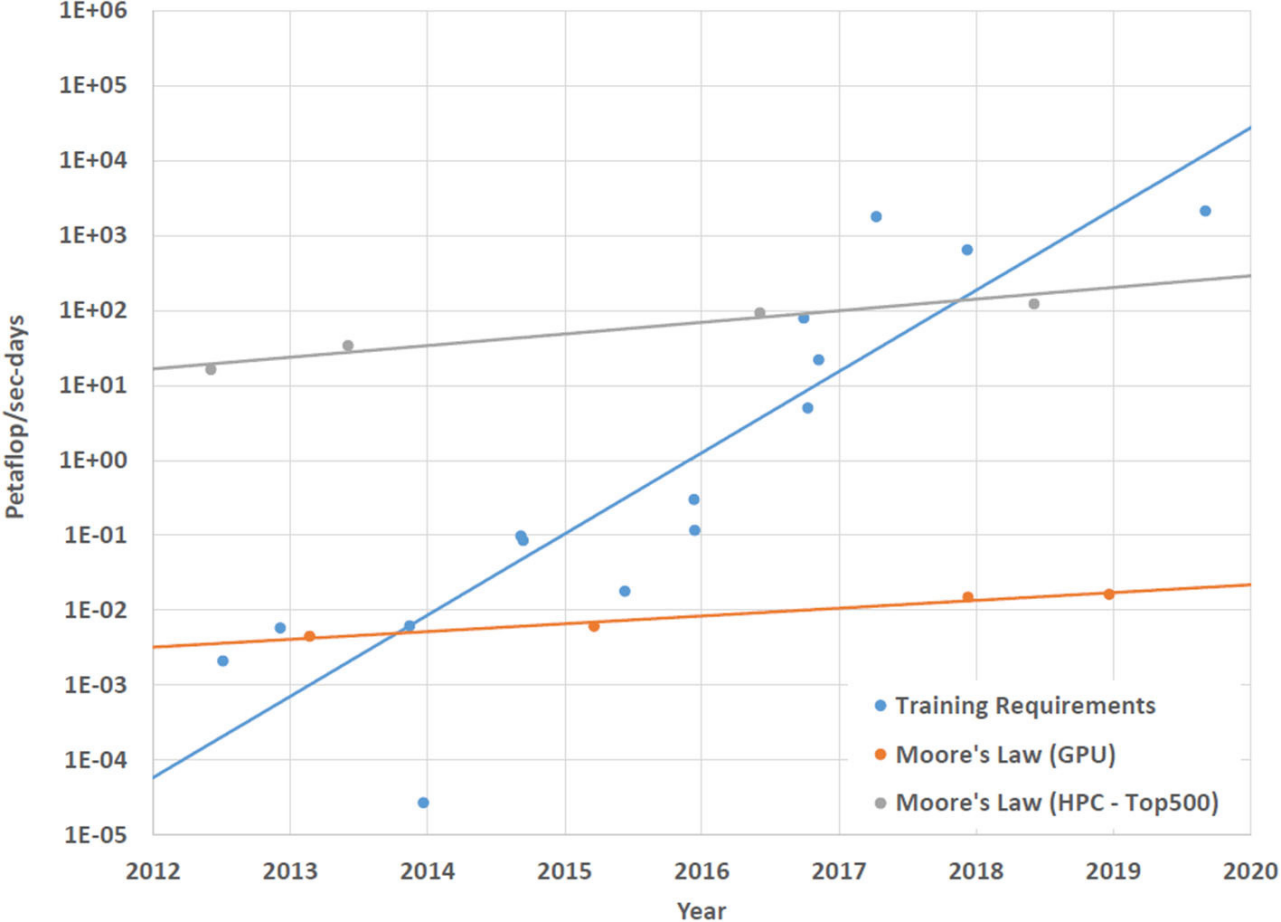
This graph shows that compute requirements for training novel AI applications is growing at a much faster rate than improvements in compute performance. This figure expands on the analysis presented in Amodei et al. (2018). The AI application data is from Hinton et al. (2012), Mnih et al. (2013), Simonyan and Zisserman (2014), Sutskever et al. (2014), Zeiler and Fergus (2014), Szegedy et al. (2015), Amodei et al. (2016), He et al. (2016), Wu et al. (2016), Zoph and Le (2016), Chollet (2017), Krizhevsky et al. (2017), Silver et al. (2017b), Silver et al. (2017a), and Vinyals et al. (2019). The GPU data is the maximum theoretical single precision floating point operations per second for the NVIDIA Titan, TitanX, TitanV, and TitanRTX GPUs. The HPC data is the maximum theoretical double precision floating point operations per second for the Sequoia, Tianhe-2, TaihuLight, and Summit supercomputers.

The amount of computation required to train more sophisticated AI applications is one of the most significant problems facing the development of these applications. As machine learning applications have advanced in terms of capabilities, their training requirements have also greatly increased. In Figure 1 we can see that over the last decade, the amount of compute required to train new machine learning applications has grown much faster than the performance improvement in computing hardware. If this trend continues, then training times for future machine learning applications will become prohibitively long and eventually untenable.

As more advanced machine learning applications are developed, understanding and incorporating more complex system dynamics will likely be required to provide novel functionality. Inspiration for the form and function of these dynamics could potentially come from biological brains as was the case with the very concept of neural networks (Sompolinsky, 2014). However, efforts to understand the purpose of biological neural dynamics are limited by the significant computational requirements of accurate models of biological neurons. Current approaches generally struggle to simultaneously support the complexity, scale, and length of the experiments that would ideally explore these dynamics.

These trends suggest that a paradigm shift is needed in the area of neuromorphic computing in order to address both the need for novel functionality and the need for improved support for training. Existing neuromorphic approaches provide a set of capabilities that include scalability, programmability, on-line learning support, complex soma models, efficiency, and accelerated simulation timescales. However, to the best of our knowledge, no architecture has been able to provide all of these capabilities simultaneously. Instead, each design has been optimized for one or more of the capabilities at the cost of others (Furber, 2016). Enabling all of these capabilities simultaneously would be a significant step in the development of neuromorphic systems that will meet the future needs of machine learning applications and computational neuroscience experiments. Superconducting electronics has the potential to support all of these capabilities simultaneously but serious challenges need to be overcome with either architecture or device solutions in order to realize that potential.

A successful superconducting architecture needs to be scalable and programmable. To satisfy these requirements, we propose a mixed-signal approach that combines superconducting digital logic with superconducting analog neuron circuits. To investigate the suitability of possible SCE mixed-signal architectures, this work presents a series of trade-off studies using numerical analysis based on measurements and designs from previously demonstrated circuits. It is important to note that this work represents an early feasibility study and that additional research is required to fully develop the architecture and ideas discussed here. This work will hopefully motivate future work in this area that will result in the development of efficient, fast, and scalable neuromorphic systems that are also programmable, biologically suggestive, and capable of supporting on-line learning. Such systems could provide a critical platform that is needed for future AI development as well as computational neuroscience research.

The main contributions of this paper are:

- A description of a novel mixed-signal superconducting neuromorphic architecture
- A detailed analysis of the design trade-offs and feasibility of mixed-signal superconducting neuromorphic architectures
- A comparison of the proposed system with other state-of-the-art neuromorphic architectures
- A discussion of the potential of superconducting neuromorphic systems in terms of on-line learning support and large system scaling.

## 2. Background

### 2.1. Neuromorphic State-of-the-Art

Neuromorphic computers are designed to replicate the structures and behaviors of the biological brain using analog or digital hardware or a mixture of both. By mimicking biology, neuromorphic computers are able to incorporate characteristics that support novel applications and research. There have been many different approaches to building these sorts of systems each with its own priorities and innovations. Five large-scale neuromorphic systems that we considered to be representative of the current state-of-the-art are compared in Table 1. This comparison is intended to be a brief sample of the current state of the field rather than a comprehensive review. As a result, there are other compelling approaches that are not included in this comparison.

**Table 1:** A comparison of representative large scale spiking neuromorphic architectures.

	**BrainScaleS**	**Neurogrid**	**TrueNorth**	**SpiNNaker**	**Loihi**
Feature size	180 nm	180 nm	28 nm	130 nm	14 nm
Neurons per core	8–512	65 k	256	1 k	1 k
Synapses per core	130 k	100 M	65 k	1 M	16 k
Technology	Analog	Analog	Digital	Digital	Digital
Soma model	AEIF	AQIF	LIF	Variable	Variable
Soma equiv FLOPS	15	12	~5	Variable (~13)	Variable (~5)
Synapse resolution	4 bits	13 bits shared	1 bit	Variable	1–64 bits
Run-time plasticity	STDP	No	No	Variable	Variable
Interconnect	Hierarchical	Tree	2D Mesh	2D Toroidal Mesh	2D Mesh
		Multicast	Unicast	Multicast	Unicast
Watts/Neuron	2.54 mW	2.31 μW	0.72 nW	62.5 μW	<5 μW
Joules/Spike	198 pJ	119 pJ	26 pJ	11 nJ	23.6 pJ

*The overall comparison expands on analysis from Furber (2016)*.

*Sources for architecture details are Rast et al. (2008), Schemmel et al. (2010), Benjamin et al. (2014), Furber et al. (2013, 2014), Merolla et al. (2014), Meier (2015), Davies et al. (2018), Lin et al. (2018), and Yang and Kim (2020)*.

*Soma Equivalent FLOPS refers to the number of floating point operations required to advance the soma model by a 1 ms time step when the model is implemented in software. This provides a very rough comparison of the complexity of the models that each architecture implements. TrueNorth, SpiNNaker, and Loihi all use soma models that incorporate varying degrees of programmability that allow for a range of model complexities. For the purposes of this comparison we assume an LIF model as the baseline configuration for TrueNorth and Loihi because that is what is reported in the literature (Cassidy et al., 2013b; Furber, 2016; Davies et al., 2018). Both approaches use a modified LIF model that would likely require more computation to simulate than a standard LIF model however it is unclear how much. SpiNNaker is assumed to use an Izhikevich model. FLOPS values are from Izhikevich (2004) and Makhlooghpour et al. (2016)*.

It is worth noting that it is possible make use of novel technologies, such as phase change memory (PCM) or Memristors to efficiently implement the functionality of different portions of the neuron (Ebong and Mazumder, 2012; Soudry et al., 2015; Sebastian et al., 2018). However, a review of the literature failed to locate an example large scale implementation of these approaches that provided the relevant details and metrics that would be needed evaluate it relative to the other approaches considered here. As a result, these sorts of approaches are not considered in the comparison in Table 1 which is focused on large scale implementations of spiking neuromorphic systems.

The primary characteristic that the approaches in Table 1 have in common is that they are designed to enable scaling to millions of neurons and billions of synapses. To achieve that scale several critical design decisions must be made regarding the complexity of the neuron model, the number and resolution of synapses to allow per neuron in the design, and the interconnect scheme used to communicate between the neurons. These decisions, in turn, affect how many neurons the system can reasonably accommodate, the power that is consumed, and the speed with which the system can update the neuron models. In some cases these decisions also limit or preclude functionality such as on-line learning (Benjamin et al., 2014).

Perhaps the most fundamental design decision of neuromorphic architectures is how much detail to support in the neuron model. It is possible to implement biologically relevant models using semiconductor electronics but this approach typically requires prohibitively complex circuit designs that have reduced yield probabilities and scaling limitations (Arthur and Boahen, 2011). An example of this trade off can be seen by comparing IBM's TrueNorth which used a completely digital design to implement a million LIF neurons on a single chip (Merolla et al., 2014) and BrainScales which used a mixed signal approach to implement a more detailed neuron model in a wafer scale system (Schemmel et al., 2010; Meier, 2015). In the case of TrueNorth, the neuron model is power and area efficient but does not capture many biological details in a single instance (Cassidy et al., 2013b). BrainScales, on the other hand, uses a more complex model but the power used by the system is significant with 1.3 W required for just 512 neurons (Furber, 2016). This is roughly 1000–10,000x more power per neuron than is needed by large neuromorphic systems that use simplified neuron models (Furber, 2016).

Another interesting design decision concerns the interconnect scheme used to enable communication between the neurons. Aspects of these schemes, such as whether the messaging is unicast or multicast and what network topology is employed, ultimately determine the bandwidth and latency characteristics of the network. These decisions also affect the complexity of the routers required to implement the network. SpiNNaker is an approach that devotes a lot of effort to solving the networking problems posed by the need for thousands of synapses per neuron (Rast et al., 2008; Furber et al., 2013, 2014). Instead of implementing the neuron model directly in hardware like the other approaches that are featured in Table 1, SpiNNaker uses a software neuron model implementation running on ARM cores that enables flexibility in terms of model choice. As a result, SpiNNaker does not directly improve the time it takes to update individual neurons relative to a software only approach. However, the innovations in the SpiNNaker network enable it to achieve impressive scales and performance despite lacking a hardware-based neuron model. Additional details regarding the design of communication networks can be found in Young et al. (2019).

Modern neuromorphic systems can achieve impressive scales and performance while using relatively little power. However, all of the current approaches have drawbacks that can ultimately be traced to technology limitations. It is possible that pursuing a neuromorphic system in a different technology, such as SCE, could open up new possibilities in terms of system capabilities.

### 2.2. Superconducting Digital Electronics Development

Recently, the field of superconducting digital electronics has been reinvigorated by the IARPA C3 and SuperTools programs. As a result, the capabilities of superconducting digital electronics have been significantly improved and are ready for use in novel architectures. In particular, the C3 program has significantly helped to drive progress in this area (Manheimer, 2015). C3 sought to address the unsustainable power demands of future CMOS-based supercomputers by developing energy efficient superconducting processors. To accomplish this goal, the program was divided into two thrusts with one focusing on developing digital logic and the other focusing on memory.

The digital logic portion of the program focused on the development of a processor with one of two competing families of superconducting digital logic: eRSFQ (Kirichenko et al., 2012) and RQL (Herr et al., 2011). The initial work of both the eRSFQ and RQL teams involved developing and demonstrating designs for basic processor components such as adders, shifters, and control logic. The eRSFQ team adopted a bit-sliced architecture for their processor while the RQL team used a more traditional bit-parallel architecture. Over the course of the program, 8-bit and 16-bit adders were successfully designed and demonstrated by the RQL team. In addition, other complex control circuits were also designed and demonstrated in RQL. These results established the feasibility of utilizing superconducting electronics to build complex, large scale processors. Designs for 8-bit and 16-bit Turing complete processors with integrated memory were developed and fabricated as part of the C3 program but are still being evaluated. Importantly, the process of developing the various digital logic designs has led to the development of design methodologies and a better understanding of how to utilize the technology to efficiently perform computations.

The memory portion of the program focused primarily on the development of magnetic memories that could provide the dense arrays needed to support larger scale computation. In general, superconducting electronics is not currently a dense technology from a fabrication standpoint. This is most obvious in the area of memory where the lack of density translates into a lack of capacity. Magnetic memories provide a solution to this because their bit cells can be potentially small and packed tightly together. Several versions of superconducting magnetic memory were investigated as part of the C3 program including JMRAM and CSHE (Ye et al., 2014; Aradhya et al., 2016; Dayton I. M. et al., 2018). In addition, to meet the immediate memory needs of the processor designs, a non-magnetic memory was also demonstrated during the program. This memory, NDRO, only utilizes JJs and so is significantly less dense than the magnetic memory alternatives. However, its characteristics made it a good fit for implementing register files and other small memory arrays that support the operation of a processor (Burnett et al., 2018).

In addition to the C3 program, other researchers have also been working to develop superconducting digital logic. One such effort that should be mentioned is using another family of logic, AQFP, to develop adders with the goal of eventually building a processor. This work has also shown promise and has had some successful demonstrations (Inoue et al., 2013; Inoue et al., 2015; Narama et al., 2015).

These developments have laid the groundwork for the development of larger, more complex digital and mixed-signal systems using superconducting electronics. Many aspects of the system architecture that is explored in this work build upon the successful demonstrations of these programs. For instance, the memory technologies developed on C3 are critical to enabling the local storage of synapse weights that are required by neuromorphic processors. Similarly, the complex control circuits that have been demonstrated are representative of the control circuits that will be needed to organize and coordinate the activities of the neurons across a neuromorphic processor.

### 2.3. Superconducting Neuromorphic Development

Many features of Josephson junctions and superconducting electronics are advantageous to neuromorphic computing. Josephson junctions have a well-defined threshold for moving into the voltage state, similar to the threshold for neurons to emit action potentials. Low-loss transmission lines can carry pulses without distortion over long distances, effectively acting as axons and dendrites. Mutually-coupled superconducting loops can weight and store circulating currents, helping to perform synaptic and summing operations. These advantages were noticed by groups in Japan in the 1990s, who proposed and measured (Hidaka and Akers, 1991; Mizugaki et al., 1993, 1994; Qian et al., 1995; Rippert and Lomatch, 1997) the first Josephson-based circuits to make simple perceptron neural networks. Work has continued since then, especially over the last 10 years or so. In this section we review recent developments, focusing specifically on the biological realism of the soma, analog synaptic weighting, and extension to larger networks.

The Hodgkin-Huxley model (Hodgkin and Huxley, 1990) is the standard for the dynamics of the action potential generated at the soma. It describes the opening and closing of sodium and potassium ion channels which allow the bi-lipid membrane of the axon to charge up (polarize) and discharge (depolarize), causing the rise and fall of the neural spike. The Josephson junction soma, or JJ soma, is a circuit of two Josephson junctions in a superconducting loop which displays very similar dynamics (Crotty et al., 2010). The two junctions act like the sodium and potassium channels, one allowing magnetic flux to charge up the loop and the other allowing flux to discharge from the loop. The result is a soma with biologically realistic dynamics that are similar to those of the Hodgkin-Huxley model.

The degree of biological realism present in a mathematical neuron model was addressed by Izhikevich (2004), who identified 20 dynamical behaviors possible for neurons. Not all behaviors are present in all neurons, but the more behaviors that a model is capable of generating, the more biologically realistic it is. The Hodgkin-Huxley model, for example, obtains 19 of the 20 behaviors. Recent work in benchmarking the JJ soma has obtained 18 of the 20 behaviors (Crotty et al., in preparation), making it more biologically realistic than alternative neuron models like the Nagumo et al. (1962) or Rose and Hindmarsh (1989). This high level of biological realism is very impressive considering it is obtained with only two Josephson junctions and the circuit is capable of running at very high clock rates (~20 GHz).

Biological synapses are responsible for coupling neurons together. They receive the action potential as their input and feed forward a small current to the downstream neuron, an action which is mediated through a chemical system of neurotransmitters. The amount of the feed-forward current is dependent on the strength or “weight” of the synapse which can, in principle, take on many values. Excitatory synapses have a positive weight and bring the downstream neuron closer to threshold; inhibitory synapses have a negative weight and push the downstream neuron away from threshold. Over time synapses can change their weight due to the coincident firing of their connecting neurons, a feature called plasticity. Plasticity allows for unsupervised training in artificial neural networks.

In order to mimic this behavior in an electrical circuit, a memory element is necessary to hold the weight of the synapse. It is possible to use a superconducting loop as this memory, holding a value of flux proportional to the synaptic strength; however, memory circuits based on loops tend have a large footprint. Following the variety of magnetic memories in the computer industry, a new kind of native synapse has recently been developed (Schneider et al., 2018a,b) using a magnetic doped Josephson junction. By putting small magnetic nanoparticles inside the insulating barrier between the two superconductors, the critical current of the junction can be changed. Since one can put many of these particles in a single barrier and they can all be in different orientations, the resulting critical current can take on essentially a continuum of values, making it an ideal memory element for the synaptic strength.

In addition to changing the critical current, these magnetic nanoparticles can alter their orientation in response to a current pulse across the junction, provided there is an external magnetic field applied parallel to the junction. This allows for the possibility of inherent plasticity: the pulsing of action potentials applied to the magnetic junction changes the orientation of the nanoparticles and hence the critical current. If this junction is embedded in a synapse circuit in which the critical current encodes the value of synaptic strength, then action potentials which arrive at this circuit will “train” the junction and alter its weight, similar to plasticity in a biological neuron.

In the analog domain, the maximum fan-in and fan-out of single neurons will be about 100 or so, limited by parasitic inductances and fabrication tolerances (Schneider and Segall, 2020). Meanwhile, in the human brain each neuron is connected to about 10,000 synapses, on average. Although it is not clear that this level of connectivity is necessary to do interesting and novel computations, increasing it above the all-electronic level of 100 is certainly worth pursuing. Toward that end, optoelectronic neurons have been recently been proposed (Shainline et al., 2016; Buckley S. M. et al., 2017; Buckley et al., 2018; Shainline et al., 2019). These neurons, operating at low temperatures, receive single photons and produce faint photonic signals. In between, photonic communication can be used to route synaptic signals over long distances through the network. Several aspects of such an integrated system have been demonstrated, including light sources, detectors and full optical links at 4K (Buckley S. et al., 2017) and passive photonic routing networks utilizing multiple planes of waveguides (Chiles et al., 2018). The superconducting neurons which interface with these photonic networks are similar kinds of Josephson neuron circuits (Shainline et al., 2019; Shainline, 2020).

In short, there is a growing “toolbox” of superconducting neuromorphic circuits which can be incorporated into spiking neural networks. One can, in fact, use these circuits on their own to design a fully-dedicated, analog neuromorphic processor. Our approach has been instead to combine some of these analog neuromorphic circuits with superconducting digital logic in a mixed-signal configuration, which we believe will ultimately result in more flexibility, scalability and programmability.

## 3. Superconducting Mixed-Signal Neuromorphic Architecture

This work builds upon recent developments in SCE and superconducting neuromorphic systems by proposing a novel mixed-signal SCE neuromorphic design: BrainFreeze. The proposed architecture combines previously explored bio-inspired analog neuron circuits with established digital technology to enable scalability and programmability that is not possible in other superconducting approaches. This is because the digital SCE components of the architecture facilitate time-multiplexing, programmable synapse weights, and programmable neuron connections. The time-multiplexing supported by the architecture allows multiple neurons in the simulated network to take turns using some of the same physical components, such as the pipelined digital accumulator. This helps to improve the effective density of the hardware. Communication between neurons in BrainFreeze is performed by a digital network like the one used by other large scale neuromorphic approaches. The digital network allows the architecture to share the wires that are used to connect the neuron cores between multiple simulated neurons. This avoids the need to provide a dedicated physical wire to connect each pair of neurons and thereby greatly improves scalability. The arbitrary connectivity provided by the digital network also allows BrainFreeze to implement a wide variety of neural network organizations by reprogramming the routing tables in the network. In this way, BrainFreeze leverages both recent developments in SCE digital logic and innovations from large scale semiconductor neuromorphic architectures to overcome the primary challenges facing SCE neuromorphic systems.

**Figure 2:**
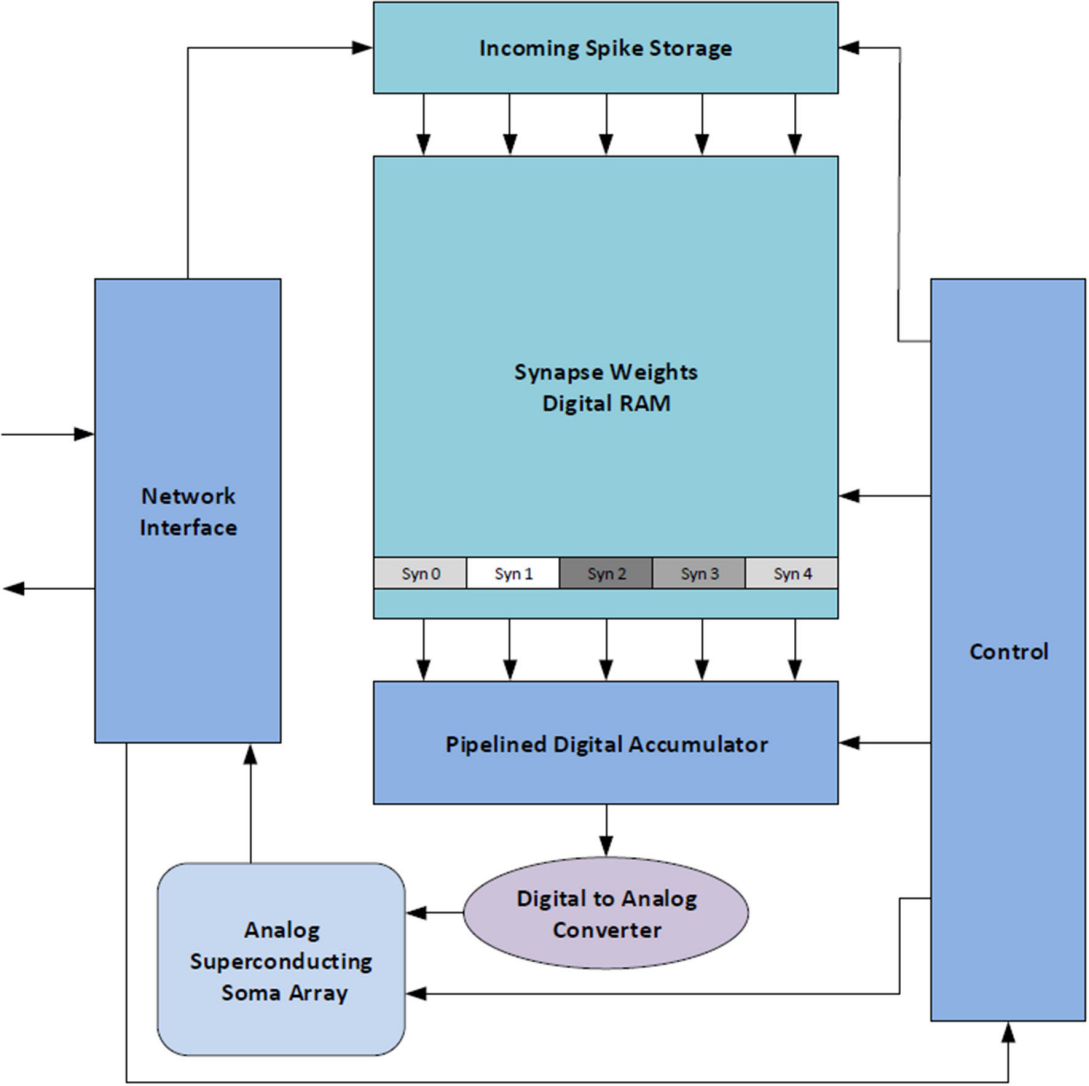
A high-level block diagram of the proposed mixed-signal SCE neuromorphic architecture that includes all of the major components. We refer to this collection of components as a Neuron Core. This architecture combines the scalability and programmability enabled by superconducting digital logic with biological suggestivity enabled by superconducting analog circuits.

The BrainFreeze architecture is comprised of 7 major components in its most basic form: control circuitry, a network interface, a spike buffer, a synapse weight memory, an accumulator, a DAC, and at least one analog soma circuit. A block diagram of the overall architecture can be seen in Figure 2. We refer to one instance of this architecture as a Neuron Core. The control circuitry ensures that the correct spike buffer entries, synapse weights and other states are used on each time step. The network interface handles address event representation (AER) (Mahowald, 1992; Boahen, 2000; Park et al., 2017) packet formation and interpretation as well as interactions with the network router. The spike buffer temporarily stores incoming spike messages until it is time to apply them to the neuron circuits. These buffers are implemented with Josephson transmission lines and should be very compact compared to other components. Multiple buffers may be needed in the case of multiplexing so that the spikes for each multiplexed neuron can be kept separate. The synapse weight memory stores the synapse weights for all of synapses implemented using the core. The accumulator is used to sum up the total incoming weight that should be applied to the neuron circuit during each time step. In order to provide a single answer as quickly as possible a tree of adders is used in the accumulator. The superconducting DAC is responsible for turning the digital output of the accumulator into the signal needed by the analog soma circuits. Finally, one or more analog soma circuits are included that can implement a biologically suggestive neuron model, such as the JJ soma (Crotty et al., 2010). These components provide the critical functionality that is required to implement a neuron. This architecture allows for the inclusion of bio-inspired analog neuron circuits in a large scale, programmable neuromorphic system.

One of the challenges that must be overcome to enable competitive large scale SCE neuromorphic systems is the limited density of SCE digital logic. SCE fabrication nodes are currently many times larger than those of CMOS. As a result, fewer SCE components can fit on a chip than is the case with CMOS. In the case of large scale neuromorphic systems this density disadvantage can result in a prohibitively small number of implementable neurons. To overcome this situation, multiplexing is used to improve the effective density of BrainFreeze's neuron core. In addition, SCE supports clock rates that are significantly faster than CMOS, so components can be multiplexed to a high degree without significantly slowing down the system relative to current neuromorphic approaches. However, multiplexing introduces latency and memory capacity requirements that must be addressed by the architecture.

Memory capacity has also been a major challenge for SCE systems in the past. This is especially concerning for neuromorphic approaches where large amounts of memory are typically needed to store synapse weights and information about the connections between neurons. To overcome this challenge, BrainFreeze leverages recently developed superconducting memory technologies and multi-temperature memory system organizations in order to ensure that sufficient memory is available to the system. In addition, BrainFreeze implements prefetching to hide some of the longer latencies associated with accessing information at different levels of its memory hierarchy.

The overall goal of the BrainFreeze architecture is to enable a combination of speed, efficiency, scalability, programmability, and biological suggestivity that is not possible in other state-of-the-art approaches. In addition, in order to help address the need for reduced training times, BrainFreeze endeavors to achieve these goals while allowing for the possibility of on-line learning support. To accomplish these goals a mixed-signal approach has been adopted but this introduces additional challenges that must be addressed by the architecture. Detailed trade-off analysis is needed to determine the best way to overcome each challenge and to establish the feasibility of the overall approach.

## 4. Methodology

The data presented in this paper is the result of detailed numerical analysis based on results from digital logic experimental demonstrations. In particular, the results are used to inform the area and latency estimates of various components of the proposed neuromorphic system. For instance, the sizes of demonstrated NDRO and JMRAM memories are used when estimating the area required for synapse storage. To estimate the area and latency of a neuromorphic core, the appropriate quantities of the component values are added together. Comparing these estimates for different configurations of the BrainFreeze architecture allows us to explore the potential trade-offs of this design. The estimates also help to establish the feasibility of the proposed architecture and provide motivation for future research.

### 4.1. Analysis Parameters

Table 2 presents the various parameters that are used throughout the analysis in this work. The memory and ALU parameters in this table come from circuits and designs that were developed for experimental demonstrations. For the purposes of this work, the memory parameters are used to estimate the area and delay required to store synapse weights and network routing information. Similarly, the ALU parameters are used to estimate the area and delay of the accumulators and control required in the BrainFreeze core. The area of an ALU is actually more than twice the area of a single adder because the ALUs included logical operations, an adder, a shifter, and control. However, this additional area is included to account for the tree of adders needed by the architecture. In other words a design with 8 parallel adders actually needs 15 adders organized as a tree to quickly produce a single result. The total area required by this tree is estimated as roughly the area of 8 ALUs or 1 ALU for each parallel adder in a design. This rough estimate is used because it is an overestimation and because the ALUs were the most suitable demonstrated designs available. The parameters for both 8-bit and 16-bit ALUs are included to show the area and latency impacts of both design choices. For this study only 8-bit adders are considered. For the purposes of this study the latencies and sizes of the DAC and soma are negligible compared to the other components of the architecture. This is possible because there are orders of magnitude fewer DACs and somas in the system than other components such as memory cells and logic gates. As a result, the impact of these components on the overall area and latency of the architecture is minimal. The MCM parameters were selected so that they could theoretically be made using a 300 mm wafer and so that 3 MCMs could fit side by side in a cabinet. The parameters for a cabinet are the dimensions of a standard server rack.

**Table 2:** The latency and size parameters that were used to generate the analysis.

	**X Size**	**Y Size**	**Latency**
NDRO (1 bit)	55 μm	55 μm	3.125 ps
JMRAM (1 bit)	5 μm	5 μm	0.25 ps (read)
			100 ps (peripheral)
8-bit ALU	844 μm	1,166 μm	550 ps
16-bit ALU	1,771 μm	2,860 μm	650 ps
Spike buffer (1 bit)	12 μm	24 μm	
MCM	200 mm	240 mm	
Cabinet (1,869 mm Tall)	600 mm	912 mm	

*The NDRO, JMRAM, and ALU parameters were taken from designs that were used for experimental demonstrations (Dayton I. et al., 2018; Hearne et al., 2018; Vesely et al., 2018)*.

### 4.2. Energy Efficiency Estimation

One of the main figures of merit (FOM) for energy efficiency in neuromorphic computing is Synaptic Operations per Second per Watt (SOPS/Watt), first introduced by IBM with their True North system. The main argument for this FOM is that it combines power and speed; a SNN which spikes twice as fast will dissipate (at least) twice as much power. For complete, working systems, this number is easily calculated by the following equation:


(1) $$\begin{array}{r} FOM=\frac{fs^{\prime }}{P} \end{array}$$

Here f is the average spiking frequency of the SNN, *s*′ is the average number of active synapses and P is the total measured power dissipated by the network. The number of active synapses *s*′ is a fraction of the total number of synapses s, typically in the 30–50% range but theoretically as high as 100%. The most efficient semiconductor SNNs achieve on the order of 10^10^ SOPS/Watt.

**Figure 3:**
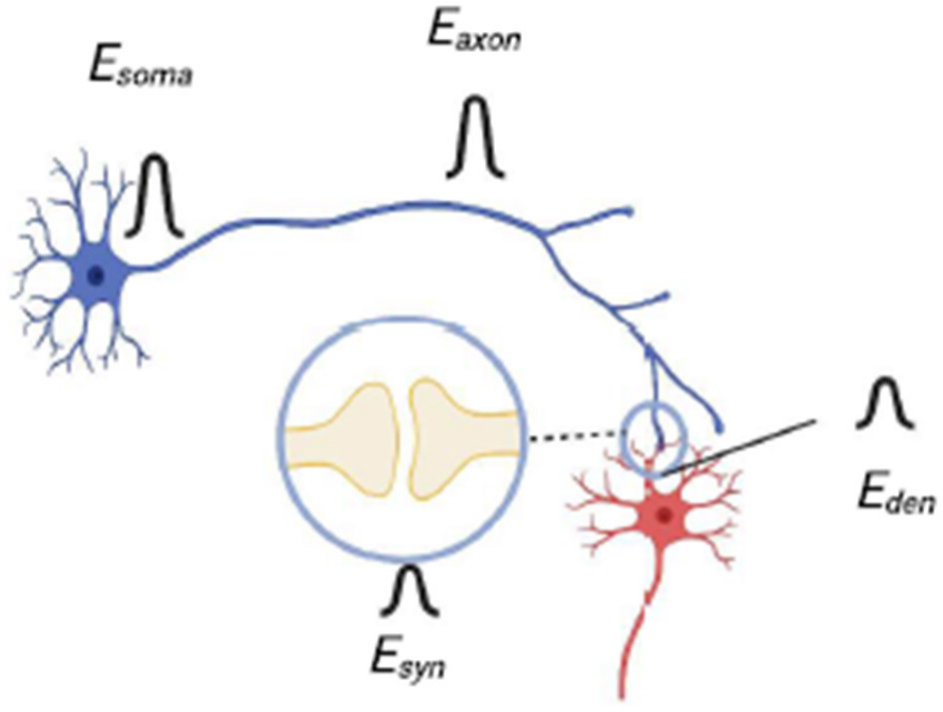
A diagram of neurons connected by a synapse that shows the placement of the components of the neurons and the corresponding energy that is associated with action performed by each component.

For systems which have yet to be fully built, we can estimate this FOM by summing up the power dissipated by the different components of the system. Figure 3 shows a schematic of a SNN showing the placement of somas, axons, synapses and dendrites. Here we assume there are N total somas and s total synapses; since there is one axon for each soma and one dendrite for each synapse, then there are also N axons and s dendrites. Let us assume that the energy dissipated per spiking event is *E*_*soma*_, *E*_*axon*_, *E*_*den*_ and *E*_*syn*_ for each soma, axon, dendrite and synapse, respectively. Then we can write the total power dissipation *P*:


(2) $$\begin{array}{r} P=fNE_{soma}+fNE_{axon}+fs^{\prime }E_{syn}+fs^{\prime }E_{den} \end{array}$$

The upper limit of P occurs when *s*′ = *s*; this is the worst-case scenario. If we assume that the energy dissipation in the axon is small compared to the soma (*E*_*soma*_>>*E*_*axon*_) and the dissipation of the dendrite is small compared with the synapse (*E*_*syn*_>>*E*_*den*_), then the second and the fourth term in Equation (2) can be ignored. Plugging into Equation (1), we then obtain (for the worst-case scenario where *s* = *s*′):


(3) $$\begin{array}{r} FOM=\frac{1}{\left(E_{syn}+\left(\frac{N}{s}\right)E_{soma}\right)} \end{array}$$

Finally, in large networks it is often true that *E*_*syn*_>>(*N*/*s*)*E*_*soma*_, since the number of synapses s are of *O*(*N*^2^). In that case FOM is simply 1/*E*_*syn*_. Of course, the above all assumes that any control circuitry or other extraneous energy dissipation in the network is either small or accounted for in one of the four energy terms; otherwise additional contributions must be added to the power in Equation (2). For BrainFreeze, we use 1/*E*_*syn*_ for our FOM; we are in the limit where *E*_*syn*_>>(*N*/*s*)*E*_*soma*_ and where our axons and dendrites, being composed of superconducting transmission lines, have negligible power dissipation. To calculate *E*_*syn*_, we count the number of Josephson junctions per synapse and assume that each junction dissipates 10^−19^ Joules per pulse (true for a junction with *I*_*c*_= 300 μA). In addition, we multiply by a factor of 500 to account for the cooling. For example, the 8-bit design of BrainFreeze has about 6,000 junctions per synapse, giving a FOM of 3.3 × 10^12^ SOPS/Watt, about 70x higher than True North (4.6 × 10^10^ SOPS/Watt).

## 5. Time-Multiplexing

One of the primary motivators behind adopting a mixed-signal approach is to enable the sharing of axon wires and core components between different simulated neurons. This time-multiplexing capability can improve scalability by allowing the system to support a larger number of virtual simulated neurons than the physical hardware would natively support. However, the improved scalability comes at the cost of increased overall simulation run-time, increased area to store the state of the additional virtual neurons, and increased complexity in terms of the organization of events in the simulated neural network. The increased control and organization complexity includes design decisions such as determining when to switch to a different virtual neuron and how to handle out of order or delayed AER packets as well as other considerations. The design and evaluation of this control logic is not relevant to the current study so we leave those tasks for future work.

The impact of time-multiplexing on simulation runtime is determined by the time it takes to update the model of each virtual neuron and the degree of multiplexing. Neuron update latency can be greatly affected by the number of inbound spikes that can be processed in parallel and the memory access delays required to access synapse weights. The number of parallel adders included in the accumulator determines how many spikes can be processed at a time. In some designs, spikes that arrive on one time step could be processed on a later time step if there is not sufficient parallel capacity to accommodate them. It is more likely, however, that such excess spikes will need to be discarded. Therefore, selecting the appropriate amount of parallelism to include affects the functionality of the system in addition to its latency. However, it is unlikely that very many of the presynaptic neurons will fire during the same time step, provided that each time step is sufficiently short. Therefore, a modest number of adders, such as 4 or 8, will likely be sufficient in many implementations of BrainFreeze. It is important to note that the latency of superconducting logic is low enough that considerable multiplexing can be employed before the simulation time step approaches that of most semiconductor architectures.

The degree of multiplexing that can be supported by the architecture is limited by the area that is available to implement the synapse memory component of the neuron core. The physical neuron hardware could potentially perform calculations related to a different virtual neuron on each time step of the system. This means that signals and states need to be preserved until the hardware is again performing calculations related to the appropriate virtual neuron. As a result, the memory system needs to have enough capacity to store all of the required information for each virtual neuron. The capacity of the memory component is directly related to its area so once the available area has been used, no additional capacity can be added to support more virtual neurons. The memory system also needs to be configured to support the number of parallel accesses required by the number of adders used by the architecture. This could be achieved by assigning particular sub-arrays of memory to each adder. In this study we assume one memory bank is provided for each adder and that the bank is sized to accommodate the number of virtual neurons present in the system.

**Figure 4:**
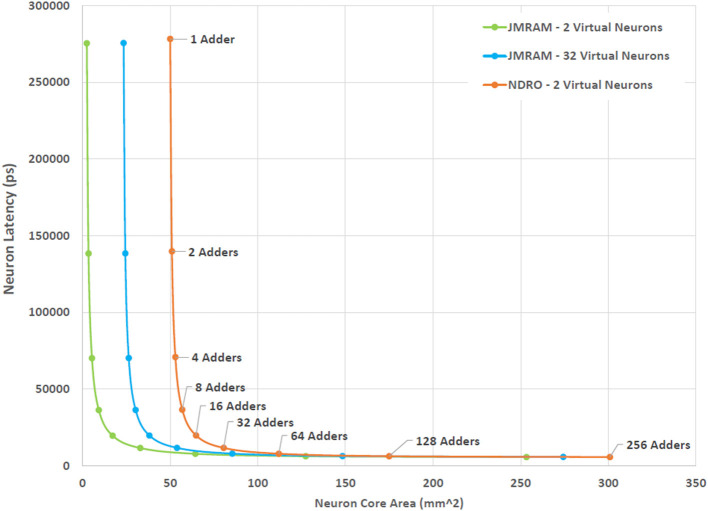
The effect of increasing the degree of multiplexing and parallelism on the area and latency of the BrainFreeze neuron core. Increasing the multiplexing improves the effective neuron density of the architecture but requires additional memory. Adding more adders enables the neuron core to process incoming spikes faster but also adds area. The latency presented here is for handling the worst case situation where all 1,000 inbound synapses receive a spike. The adders used in this analysis are 8-bits wide.

Figure 4 illustrates the trade-off that exists between the degree of multiplexing, area and latency in the design space of the neuron core. The trade-off shown in Figure 4 captures the worst case scenario for a neuron core where a spike is received on all 1,000 synapses in the same time step. The actual number of spikes that may be seen per time step is a function of design decisions that are beyond the scope of this study. As a result, selecting the optimal number of adders for BrainFreeze is a subject for future work. For the purposes of this study the highly unlikely worst case is used as a stress test to evaluate the impact of neuron update latency. We use 8-bit adders for this trade-off analysis because the synapses in our proposed system have 8 bits of resolution. In this figure we can see that the area impact of increasing the number of adders is minimal until 32 or more adders are used. Prior to that point, the area is primarily affected by the degree of multiplexing due to the memory that is required to store the synapse weights of the additional virtual neurons. The impact of memory technology choice is emphasized by these results as just 2 virtual neurons worth of synapses require more area in NDRO than 32 virtual neurons required in JMRAM. These results suggest that 16 adders may represent a reasonable upper bound design point for a 1,000 synapse BrainFreeze system. A design with 16 adders provides coverage for many simultaneous spike events but does not require too much area or introduce too much delay. A smaller number of adders may be more reasonable if smaller numbers of spikes per time step is typical in a system or if update speed is not the most important design aspect.

## 6. Synapse Density

A primary metric of feasibility for SCE mixed-signal neuromorphic designs is the number of synapses that can be implemented per neuron using this approach. The quantity and resolution of the synapses that a neuromorphic architecture can implement can greatly affect the types of networks that can be implemented on that architecture and the performance of those networks. Efficiently ensuring the largest number of synapses, each with an acceptable resolution, is a major consideration of all neuromorphic architectures. This is particularly true in superconducting neuromorphic systems where the memory capacity required to store synapse weights is at a premium. The sizing and organization of the synapse memories need to be balanced to provide the maximum number of synapses possible without negatively affecting other aspects of the neuron core design. For this early study of the potential of BrainFreeze, we have settled on an 8-bit synapse resolution. 8-bits is more resolution than is used by many other neuromorphic architectures and provides a wider range of synapse weights.

Synapse latency can be a major contributor to overall core latency. If the memory arrays are too large they can incur very long latencies which will negatively affect performance. In order to minimize synapse weight access latency while providing adequate storage capacity, the memory will likely need to be organized into many banks. Each bank will then hold a subset of the total synapse population for each virtual neuron. This organization introduces the issue of bank collisions where several synapse weights that all map to the same bank are needed at the same time. Improperly handling this situation could lead to a degradation in performance or functionality as spikes will either be delayed or discarded as a result of the collision. One possible solution to this issue is aggressively banking the memory array such that each array is so small that very few synapses for a particular virtual neuron will map to the same bank. This would greatly reduce the odds of a collision but would introduce area and delay overheads due to the additional hardware required to interface with and manage so many banks. For the purposes of this study we consider only the area required for the memory to support some number of neurons.

**Figure 5:**
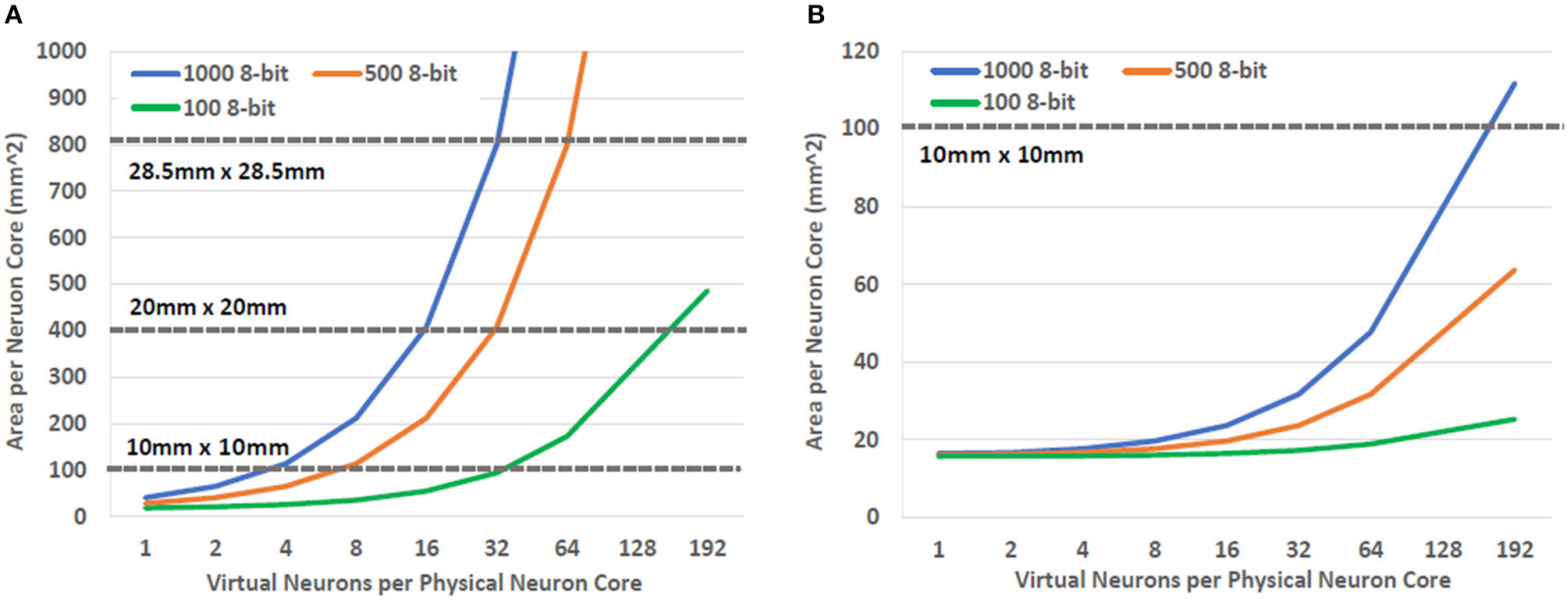
The die area required to support BrainFreeze neuron cores as the number of synapses per neuron and the degree of multiplexing is increased. These results show that **(A)** NDRO and **(B)** JMRAM based designs have sufficient capacity to act as local buffers for neuron information. Both NDRO and JMRAM also provide enough capacity to directly support small scale and larger demonstrations, respectively.

In order to determine how many synapses could be accommodated on typical die sizes, we calculated the area required for a neuron core as the number of virtual neurons per core was increased. The area needed for other components of the neuron core was also included in this calculation. For this study a 16 adder configuration was used based on the result presented in Section 5. The results of these calculations can be seen in Figure 5. Here we can see that both NDRO and JMRAM can support more than one virtual neuron using typical die sizes. In fact, hundreds of thousands of synapse weights can fit on a single die when a relatively dense superconducting memory technology, like JMRAM, is used to store them. Nevertheless, even less dense technologies, such as NDRO, still enable synapse counts that are appropriate for near term demonstrations. Importantly, these memories provide sufficient capacity to act as local buffers for systems that use a backing store to enable even larger numbers of virtual neurons. The implications of this capability are discussed later in Sections 8 and 9.

## 7. Network Configuration

Programmability is another important aspect of a neuromorphic architecture because it allows the system to implement different neural networks using the same hardware. A key aspect of programmability is that the connections between neurons need to be configurable so that a wide variety of neural network topologies can be implemented. This can accomplished by implementing the connections between neurons as a computer network using routers and shared wires. Each neuron is given a network address and spike messages are routed through the network based on those addresses. AER representation could be used such that each spike message contains all the information needed by the post synaptic neuron core to apply the spike to its current state. This information could be as simple as indicating the presence of a spike on that synapse during this time step or could include finer grained temporal information or other spike characteristics.

The architecture of the digital network between the neurons greatly affects the functionality and speed of the overall network. In general, there are three primary decisions regarding the network architecture of neuromorphic systems: the network topology, the packet type, and the routing style (Young et al., 2019). The topologies that are often employed by state-of-the-art neuromorphic architectures are the 2-D mesh and the tree. Of these two topologies, the 2-D mesh topology provides better bisection bandwidth due to the larger number of links in the topology compared to a tree topology (Benjamin et al., 2014). However, the tree topology generally has fewer hops between destinations than the 2-D mesh and so it provides better latency. An important aspect of both of these topologies is that they can be implemented using routers with a relatively small number of ports (radix). The radix of the router (number of ports) greatly affects its complexity and increasing the number of ports rapidly grows the size of the router.

**Figure 6:**
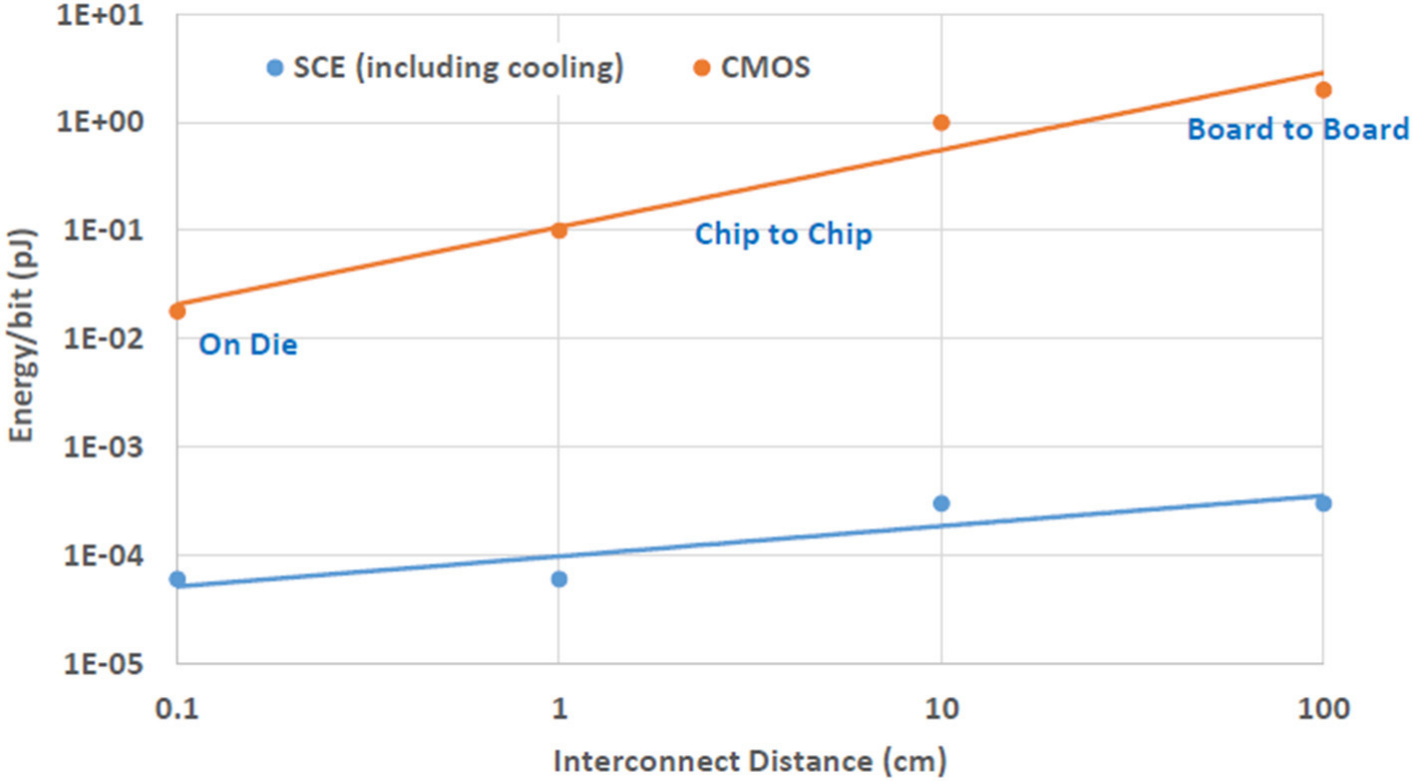
The energy cost of communicating across different distances in both CMOS and SCE. The efficiency of SCE wires provide a distinct advantage that can be leveraged by networks of BrainFreeze neuron cores. The CMOS data in this figure is from Borkar (2011).

A tree topology seems to be a good design choice for SCE neuromorphic systems. The limited density of SCE means that topologies that require higher radix routers are probably not a reasonable design choice at this time. Therefore, the ability to implement a tree topolgy using low radix routers satisfies an important requirement of BrainFreeze. In addition, the superconducting wires used to communicate in BrainFreeze provide a potentially useful advantage over room temperature wires in terms of their efficiency over long distance. A comparison of the energy required for long distance communication using both traditional semiconductor as well as superconductor wires can be seen in Figure 6. This difference in communication energy means that topologies that feature longer connections may be better suited to implementation in SCE-based systems. So, the longer connections that can exist in a tree provide additional motivation to use this topology in an SCE-based system like BrainFreeze.

In addition to the topology of the network, designers must also determine what style of packets to use and how to route them. Packets can be either unicast or multicast, meaning that one packet can either have one destination or many. Neural spikes tend to have multiple destinations so a multicast network seems to make the most sense. Networks that support multicast packets can maximize bandwidth efficiency by only generating additional packets at the routers that have destinations on more than one port. Source address routing can help to minimize the logic required in the router to provide this functionality. However, in order to support source address routing a large memory is required in the router to store all of routing information for each source address. Destination address routed packets, on the other hand, include the connectivity information in the packet itself. So they can be implemented without the need for a large memory in the router. These additional requirements in terms of memory and router complexity mean that the multicast and source routed approaches would require routers that would be prohibitively large and slow in an SCE neuromorphic system. As a result, a unicast, destination routed network is likely the best choice when only on-die memory is available to the router.

**Figure 7:**
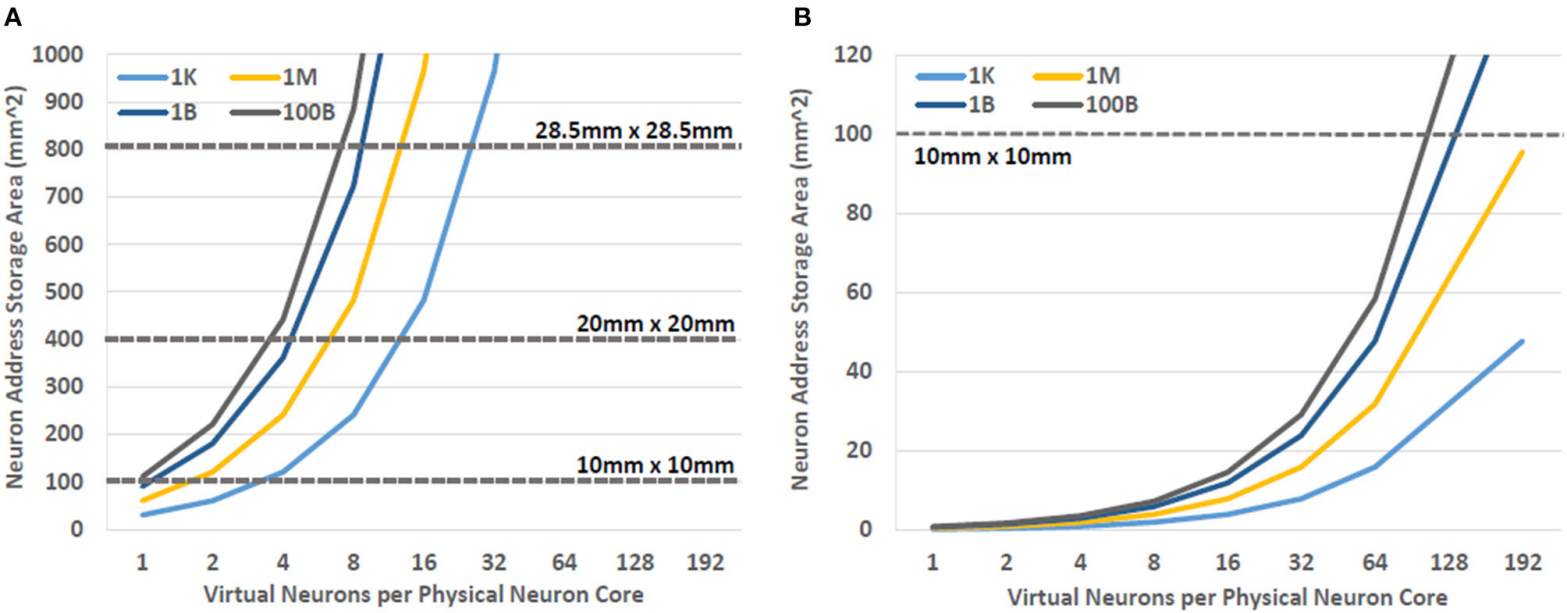
The area required to store the post-synaptic addresses for neural networks with increasing numbers of neurons. As the network grows the area required to store this information can become prohibitive. The incorporation of a backing store may be an effective way of addressing this need for additional memory capacity. **(A)** NDRO and **(B)** JMRAM.

Even in a unicast, destination routed network, the memory requirements of the destination neuron address storage quickly dominate the overall memory requirements of the architecture as the size of the network grows. In other words, as the network size grows to millions of neurons or more, we need more memory to store the addresses for the post synaptic neurons than we need to store the synapse weights. This can be seen in Figure 7 where the area required to store neuron addresses increases rapidly as the size of the overall simulated neural network grows. Therefore, JJ-based memories, such as NDRO, are likely only sufficient to provide storage for both synapse weights and post-synaptic addresses in networks that contain a few thousand neurons or less. JMRAM can support larger numbers of neurons but would require a prohibitive number of chips to support the hundreds of millions of neurons that some state-of-the-art approaches can support. The addition of a backing store to the system could alleviate this situation by providing additional capacity for address storage for very large systems. Furthermore, a backing store might also enable support for multicast, source routed networks by providing sufficient capacity for the larger routing tables that that communication scheme requires.

## 8. Memory Hierarchy

**Figure 8:**
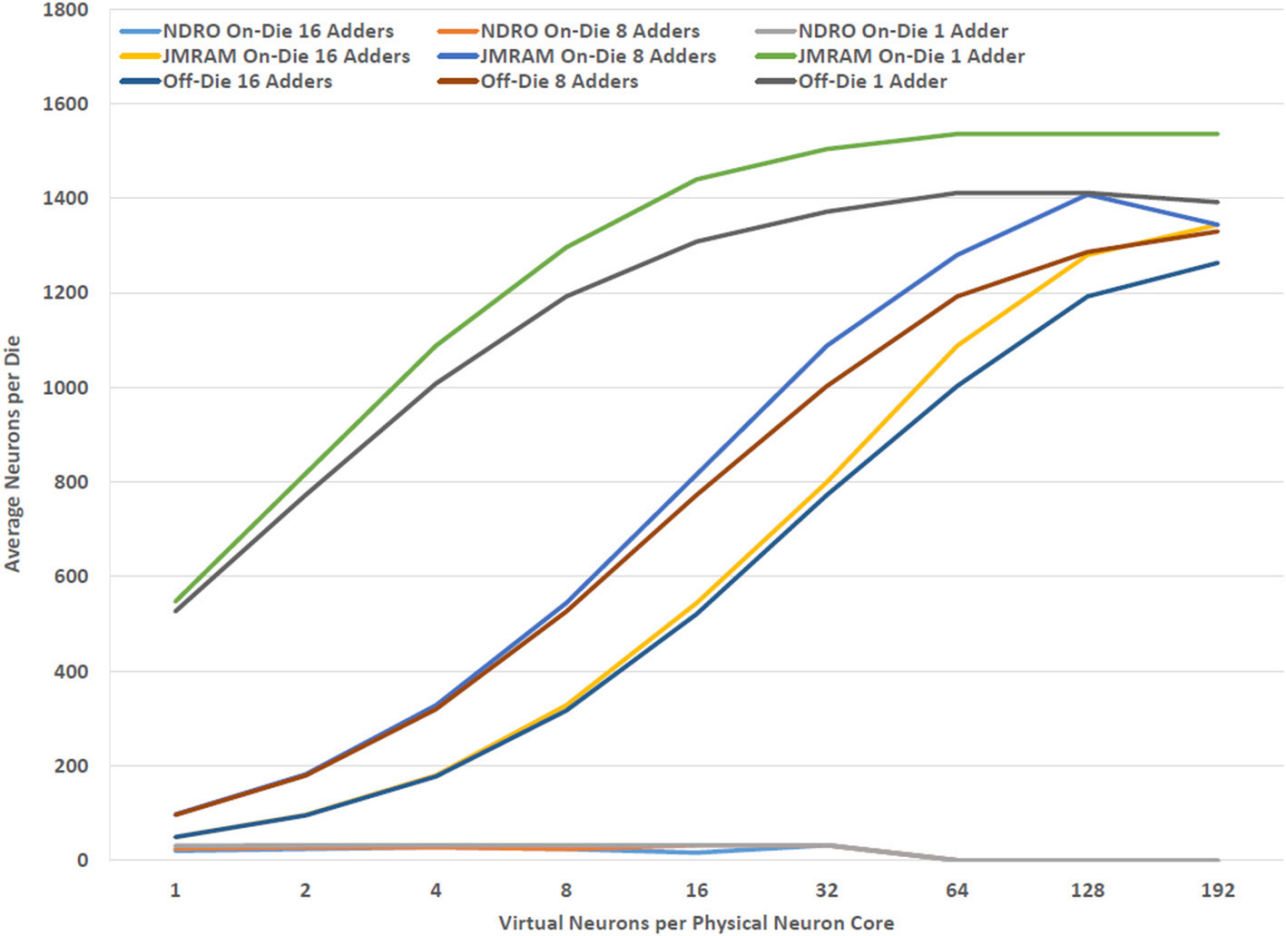
The relationship between effective neuron density and the location and type of memory. Using separate dies for JMRAM does not improve the number of neurons that can be implemented per die in BrainFreeze.

Providing enough memory to meet the requirements of each component in the BrainFreeze architecture is particularly challenging due to the relatively limited memory density available in SCE memory technologies. One solution is to include chips in the architecture that serve as off-die memories for the neuron cores. Theoretically, these off-die memories would free up space on the neuron chips so that more neuron cores could be implemented on those chips. Similarly, the dedicated memory chips should provide additional memory capacity because the memory arrays should fit more tightly together. This approach also has the benefit of easing any integration issues that might arise from incorporating magnetic memories and SCE logic circuits on the same die. However, as Figure 8 shows, placing neuron cores and synapse memories on separate chips does not actually result in improved neuron core density. This is because removing the memories from the neuron chip does not free up enough space for additional neuron cores to offset the cost of adding the memory chips to the system. Clever floorplanning of neuron cores could potentially address this though that is beyond the scope of this study.

**Figure 9:**
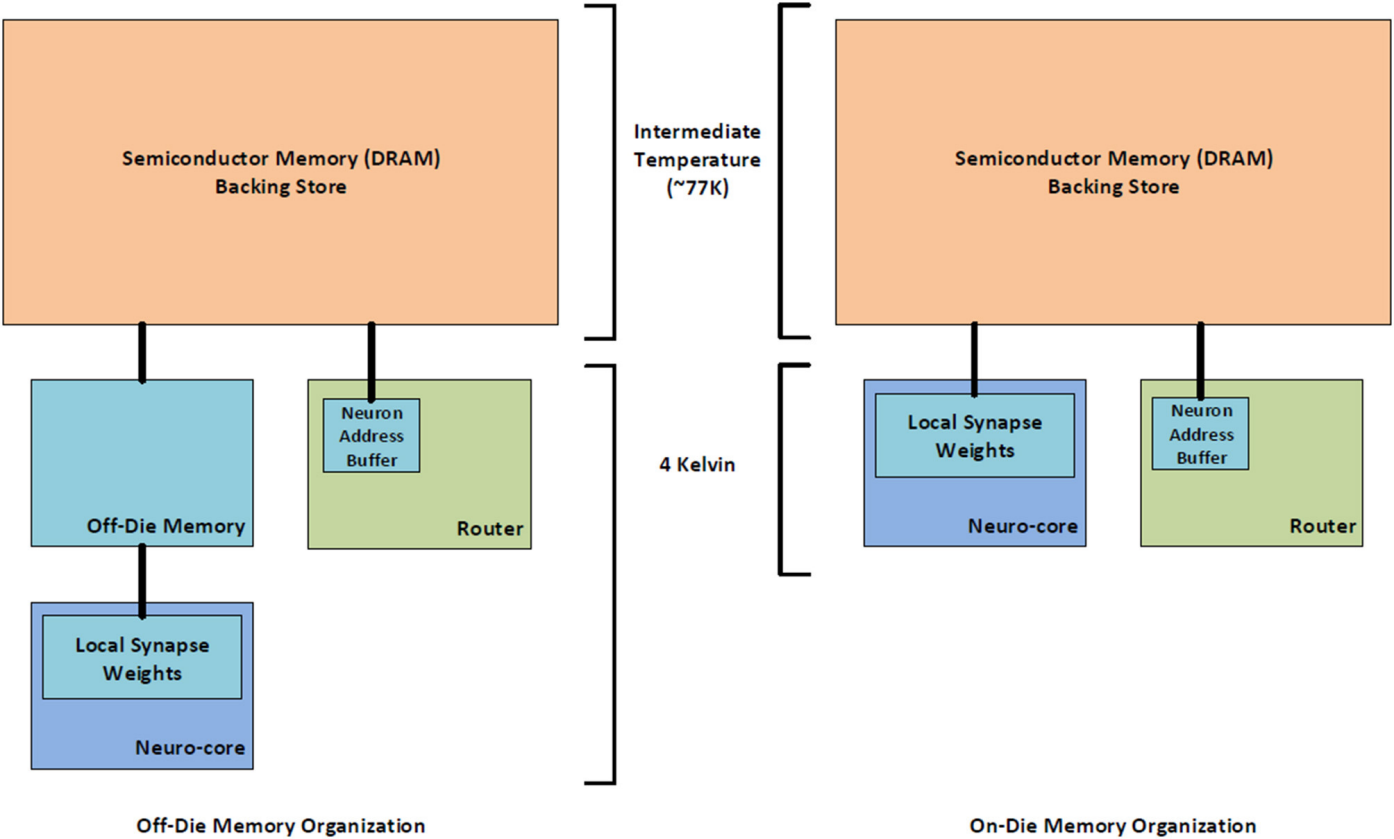
A block diagram of the proposed multi-temperature memory system that would enable the use of cryo-DRAM with the BrainFreeze architecture. This could greatly increase the memory capacity available to BrainFreeze.

The available on-die memory is sufficient for storing the synapse weights for a reasonable number of virtual neurons. However, the limited capacity of superconducting memories will ultimately limit the number of neurons and synapses that can be implemented using only superconducting electronics. To achieve continued scaling, denser memories are needed. One solution to this problem may be a multi-temperature memory hierarchy that uses semiconductor memory technologies as a backing store for the superconducting memories. An example of such a hierarchy can be found in Figure 9. The much denser semiconductor memories enable an overall memory capacity for the superconducting architecture that is comparable to the memory capacities of semiconductor approaches. To alleviate some of the power and heat considerations that would be introduced by adding a large amount of semiconductor memory to the SCE system, the semiconductor memory could be implemented at an intermediate temperature. Cryogenic-DRAM is a technology that could potentially be used to fill this role in the system (Tannu et al., 2017; Ware et al., 2017; Wang et al., 2018; Kelly et al., 2019).

Local superconducting memories will still needed to buffer the data because there is a considerable latency involved with accessing data stored in the semiconductor memories. These local buffers could be much smaller because they would only need to contain a subset of the total system data. This could improve neuron core density, increase the number of synapses that could be implemented per virtual neuron, and result in much better area utilization in off-die memory organizations. In practice, the amount of data that needs to be buffered locally will depend on the type of data. For instance, only the post-synaptic neuron addresses for the current virtual neuron would need to be buffered. However, if the system is supporting spike time dependent plasticity (STDP), then the buffer would need to be large enough to store the synapse weights for multiple virtual neurons. This is because STDP may result in synapse weight changes after a virtual neuron has completed its update. In both cases the buffer needs to have room to store the incoming data that will be used by the next virtual neuron to be handled by the neuron core. So in the address case, the buffer would need to hold two virtual neurons worth of data and in the weight case it would need to hold three or more virtual neurons worth of data. This is still considerably less than the amount of memory that would be needed to store all of addresses and weights for all of the virtual neurons that are assigned to a neuron core. However, the latencies involved with requesting data from the intermediate temperature backing store are prohibitive. A technique is needed to improve or hide these latencies in order for the multi-temperature memory hierarchy to be a viable solution.

## 9. Prefetching

One way to hide the latency involved with retrieving data from a memory is to request it in advance of when it is actually needed. That way the data can arrive just when it is needed and the latency involved will not affect the performance of the system. This technique is called prefetching. The problem with prefetching is that it's not always easy to know what should be requested from memory. As a result, prefetching can sometimes result in performance degradation as it can tie up memory resources with requests for data that will not be needed anytime soon. Therefore, the key to effectively utilizing prefetching is correctly anticipating which data will be needed in the near future.

Neuromorphic systems like BrainFreeze are particularly well suited to prefetching because their data accesses follow predictable patterns. This makes determining which data will be needed in the near future relatively straightforward. For instance, the addresses and weights that are needed for the next virtual neuron can be fetched while the current virtual neuron is being updated. This deterministic behavior ensures that the data that is prefetched, is data that is likely to be needed. As a result, prefetching can provide an effective way to hide the latencies incurred by using a multi-temperature memory hierarchy. This allows BrainFreeze to preserve its performance advantage even when using a longer latency backing store.

**Figure 10:**
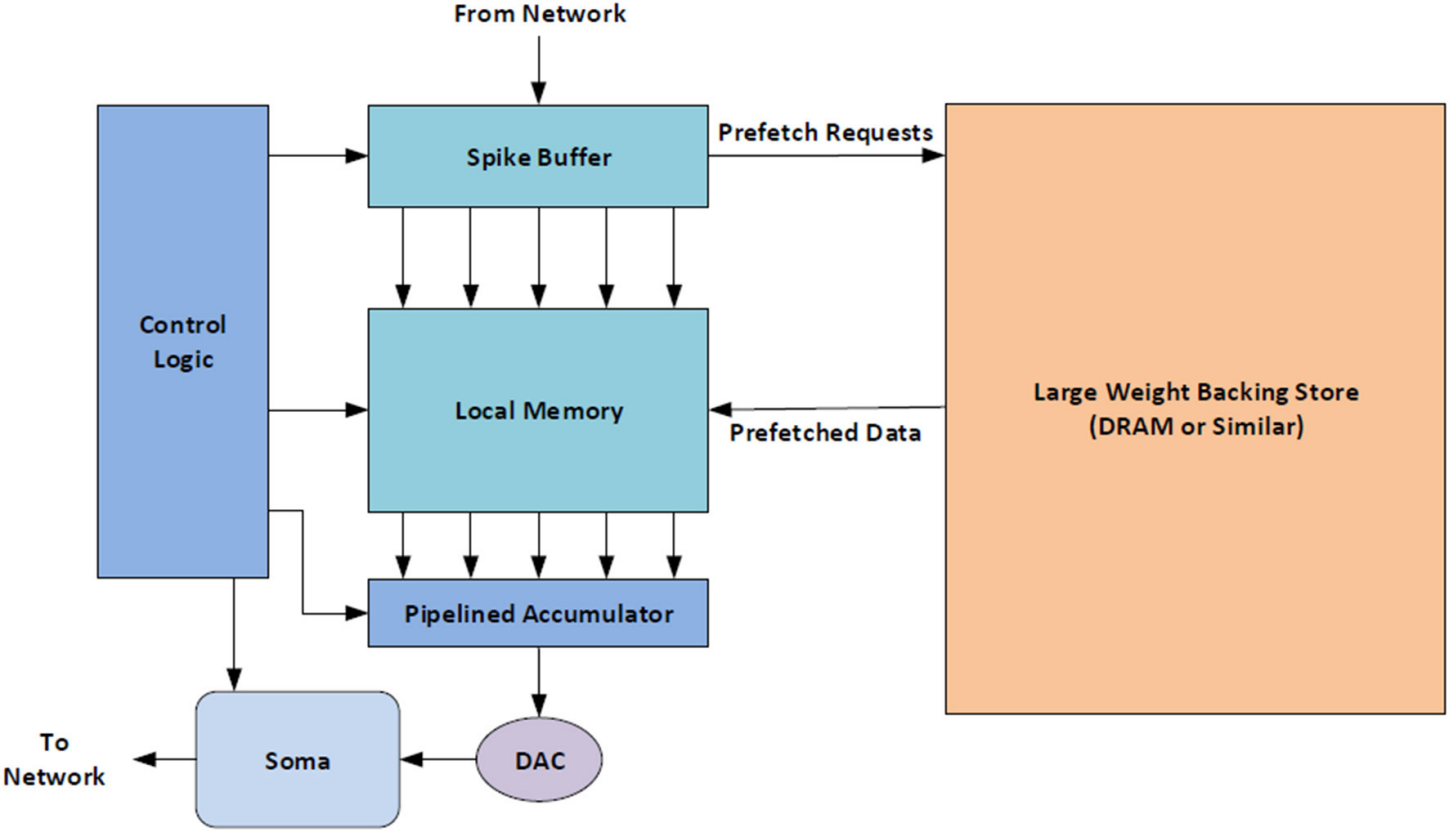
A block diagram of the proposed prefetching system that would use information in the neuron core, such as which synapses received a spike, to better avoid fetching data that will not be needed.

Of course, not all of the relevant addresses or weights are always used during the update of each virtual neuron in the network. As a result, some data will be fetched that is not used and therefore it is possible that some energy will be wasted with this approach. One way to avoid this problem is to use more information from the system to determine exactly what data should be prefetched. For example, the spike buffer holds a record for each inbound spike that will be applied to a virtual neuron when it is next updated. These records can be utilized to specify a subset of the synapse weights to read from the backing store rather than requesting all of the synapse weights that might be needed by a virtual neuron. In this way, unnecessary data accesses due to prefetching can be greatly reduced and degradation to memory efficiency and performance can be avoided. A block diagram of a potential implementation of this scheme is provided in Figure 10.

## 10. Comparison to State-of-the-Art

Now that we have established some feasible design choices for the BrainFreeze architecture, it's important to evaluate how that architecture might compare against other state-of-the-art approaches. For the purposes of this evaluation we compare against the neuromorphic architectures that were described in Section 2.1. These architectures represent some of the most successful and studied neuromorphic designs that have been proposed. Details regarding the methodology used to produce these comparisons can be found in Section 4.2 of this paper. This analysis attempts to compare only the neuron implementations of each approach. As a result, only the components of the BrainFreeze neuron core are included in these calculations. Other potential supporting hardware, such as a cryo-DRAM backing store, are not considered. To the best of our knowledge, the values used for other neuromorphic approaches also do not include supporting hardware.

**Figure 11:**
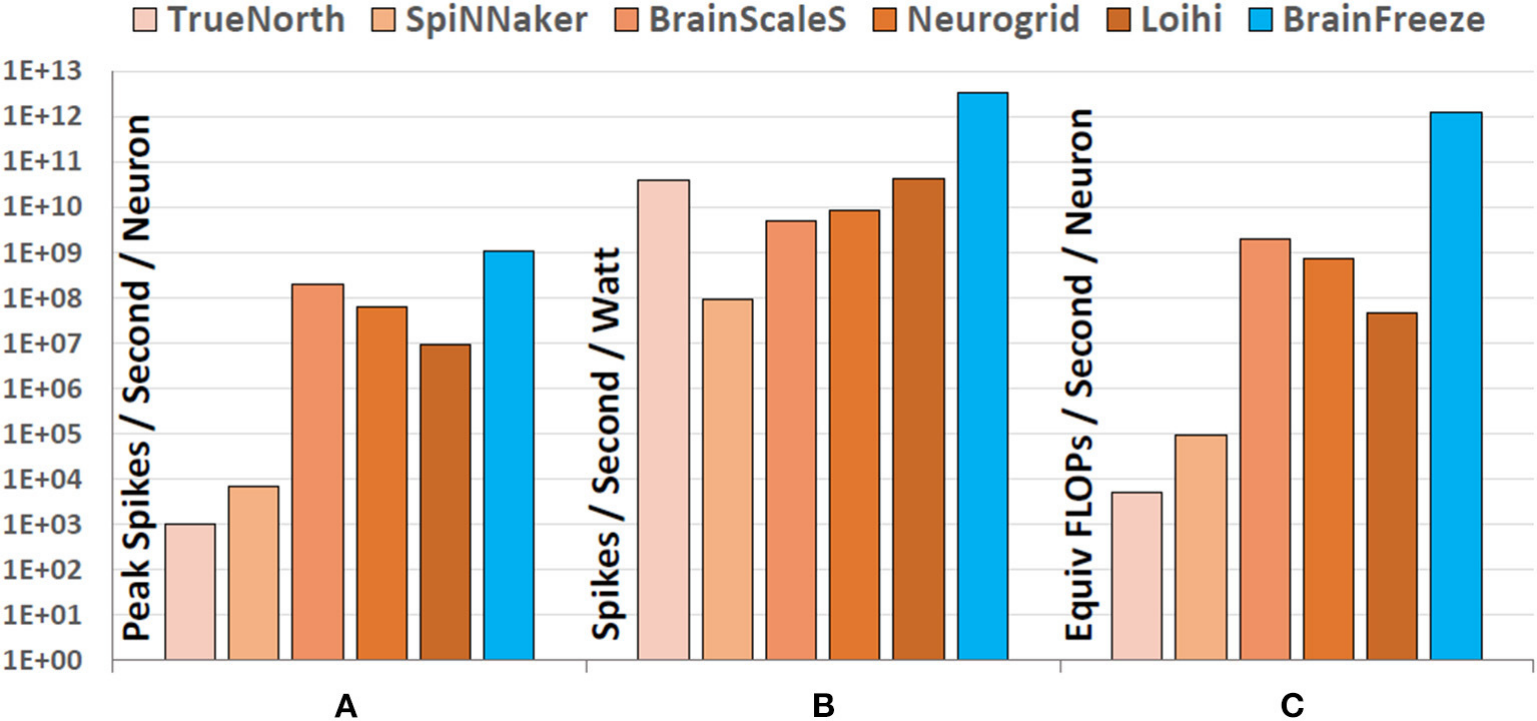
A comparison of state-of-the-art neuromorphic architectures to BrainFreeze in order to demonstrate the potential of the proposed architecture. **(A)** The first comparison looks at how quickly each design can update its neuron model and emit a spike. **(B)** The second comparison looks at how efficient each architecture is at performing its computations in terms of time and power. **(C)** The third comparison looks at the computational complexity of the neuron models implemented by each architecture. Overall, BrainFreeze have the potential to enable significant improvements over current neuromorphic approaches.

One of the most compelling capabilities of SCE is its ability to support very high clock rates. To determine how this might benefit a neuromorphic system we consider the peak rate at which neuron updates might occur in each of the different architectures. For the purposes of this study we consider such a neuron update to be roughly equivalent to the process it takes to produce a spike in response to incoming signals. The peak spike rate is one of several factors that can affect how quickly a simulation can be performed by the hardware. The values in Figure 11A for peak spike rate where determined by consulting the literature to determine the rate at which each established architecture can produce a spike (Benjamin et al., 2014; Stromatias et al., 2015; Amir et al., 2017; Davies et al., 2018). For TrueNorth the system clock frequency is used to estimate the maximum spike rate and for Loihi the mesh-wide barrier synchronization time is used. This peak spike rate is calculated on a per-neuron basis, rather than per-chip, to provide a direct comparison between the designs. The peak spike rate for BrainFreeze was calculated for the ideal situation where only a single action potential arrives and only a single adder is needed for its integration. The spike rate for BrainFreeze includes the latency for each of the major components of the SCE neuron core including memory lookup and digital accumulation. The results of this comparison show that, like BrainScaleS, BrainFreeze is capable of running significantly faster than biological real time. This is an important result as it indicates that the high clock rates enabled by SCE will support the accelerated simulation time scales that will likely be necessary for future AI research.

However, speed alone cannot meet all of the anticipated requirements of future AI research. Efficiency is another important aspect of neuromorphic systems as it can ultimately be a limiting factor on the size of the system that can be reasonably implemented. As a result, very large simulations require efficient neuromorphic systems to effectively host them over acceptable time scales. From the comparison in Figure 11B. we can see that some approaches, such as TrueNorth, use extremely efficient hardware to achieve impressive overall energy efficiency despite the relatively slow spiking rate of the hardware. Other approaches, such as BrainScales, are capable of achieving extremely fast spike rates and accelerated simulation times but pay a heavy price for this speed in terms of power so their overall energy efficiency is not as good as other designs. Balancing between speed and efficiency often results in designs that deliver impressive overall energy efficiency but that cannot simulate faster than biological real-time. For most of the past neuromorphic approaches this was acceptable since simulating faster than biological real time was not a primary design goal. BrainFreeze is capable of simultaneously delivering both speed and energy efficiency that is nearly two orders of magnitude better than current state-of-the-art approaches. This is possible because of the low power characteristics of SCE.

A discussion of the computational efficiency of the SCE approach should also take into account the complexity of neuron model that the approach enables. This is an important point of comparison against existing neuromorphic designs because all of them implement less complex models such as LIF, AdEx or the Izhikevich model. If these designs instead implemented the Hodgkin-Huxley model then it is reasonable to assume that their energy efficiency would greatly decrease either due to the extended neuron update times or due to the power needed to support additional hardware. The ability of BrainFreeze to support a biologically suggestive model similar to Hodgkin-Huxley means that each neuron update is not only happening in less time, it is also more computationally complex. To illustrate this difference in complexity between the neuromorphic approaches we compare the effective FLOPs/Sec. These values are based on the FLOPS per 1 ms simulation time step values that are provided by Izhikevich in Izhikevich (2004). Here we take the 1 ms simulation time step to be roughly equivalent to a neuron update in each of the neuromorphic designs. So, to estimate the equivalent simulation work that is being accomplished by each architecture per second, the FLOPS value is multiplied by the spike rate for each approach. This comparison can be seen in Figure 11C. The ability of BrainFreeze to support a model similar to Hodgkin-Huxley means that each neuron update is roughly equivalent to 1,200 FLOPs in a software implementation of the model. This effect combined with the speed of BrainFreeze results in an improvement of nearly three orders of magnitude compared to the other designs.

Overall these results show that a mixed-signal SCE neuromorphic approach could provide significant improvements over the current state of the art in terms of speed, energy efficiency, and model complexity. These improvements are important because they will enable future neural network simulations to run in less time while simultaneously incorporating more biologically inspired functions.

## 11. On-line Learning Support

Improving the computational efficiency of neuromorphic systems will help to address some of the needs of future neural network simulations but training times also need to be accelerated. Figure 1 in Section 1 illustrated the immediate need for enhanced training methodologies as training requirements are growing much faster than the capabilities of computer hardware. One way to approach the problem of computationally intensive training is to use on-line learning to perform at least some of the training. On-line learning provides three potentially useful capabilities to a neuromorphic system like BrainFreeze. First, the improved speed and efficiency of BrainFreeze could possibly result in on-line learning reaching an acceptable solution in less time and using less power than other approaches. Second, utilizing on-line learning could allow the implemented neural network to adapt to changes in the input data thereby avoiding the need to completely retrain the network. And third, on-line learning is more biologically relevant and may help to support computational neuroscience experiments. For these reasons incorporating on-line learning into neuromorphic systems could prove to be a valuable capability for future applications and experiments.

On-line learning techniques train the neural network using a stream of input data rather than training the network with an entire set of data prior to run-time. In spiking neuromorphic systems like BrainFreeze, on-line learning techniques can take the form of run-time synaptic plasticity where synapse weights are adjusted as neurons in the system react to inputs. One on-line learning technique that may be particularly well suited to SCE neuromorphic systems is Spike-Time Dependent Plasticity (STDP). STDP is a Hebbian reinforcement learning rule that updates the synaptic weights based on the timing relationship between the input and output spikes of neurons. Hebbian learning seeks to increase the synaptic weight of a synapse if the pre-synaptic neuron tends to influence the firing of the post-synaptic neuron. In STDP this influence is determined by considering the timing of the firing of pre and post synaptic neurons. If the pre-synaptic neuron tends to fire before the post-synaptic neuron then the weight of the synapse between them is increased. Conversely, if an action potential from the pre-synaptic neuron does not typically result in an output from the post-synaptic neuron then the weight of the synapse between them should be decreased.

**Figure 12:**
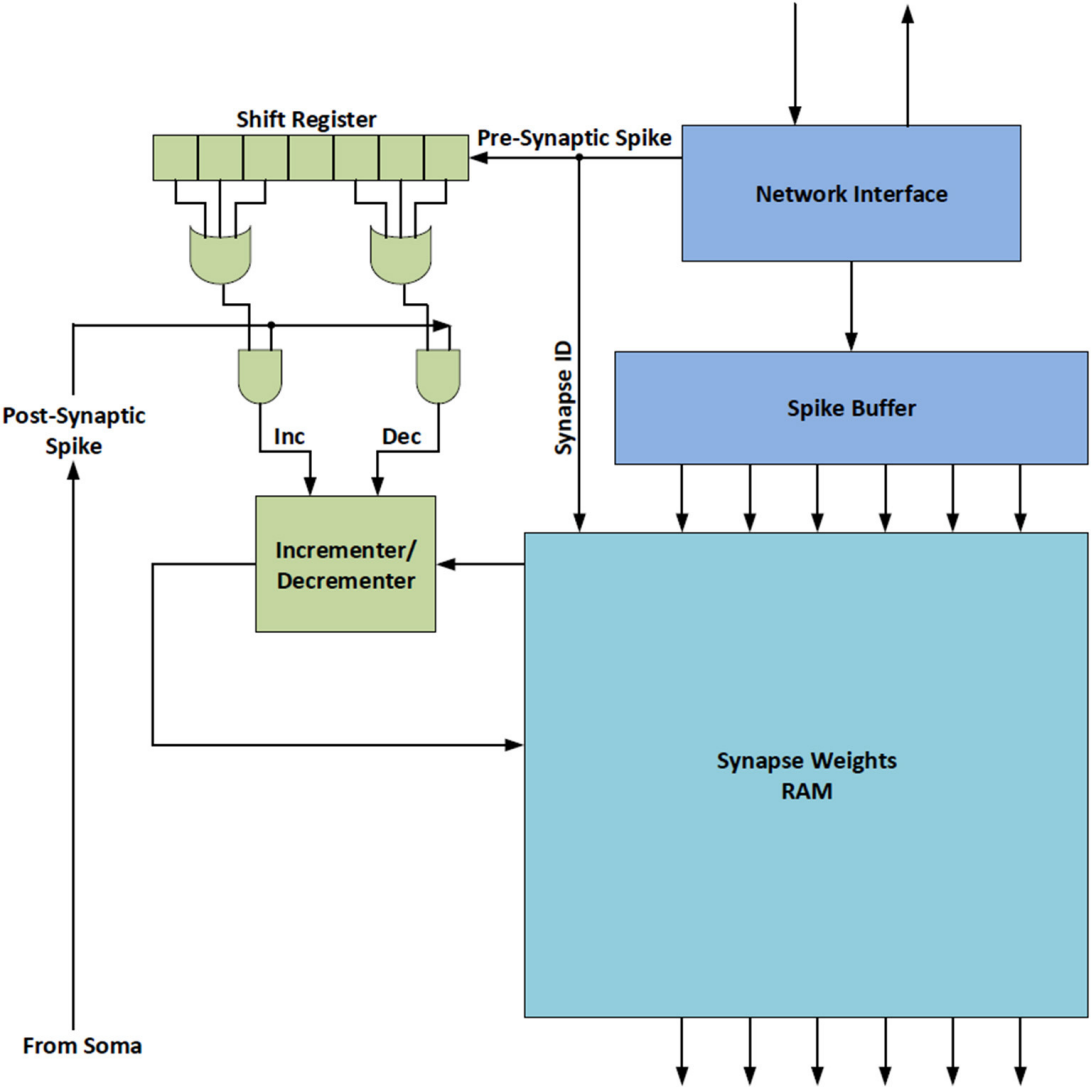
A block diagram of an example hardware implementation of Spike-Time Dependent Plasticity (STDP) that was inspired by Cassidy et al. (2013a). This hardware could allow BrainFreeze to support on-line learning.

In order to incorporate on-line learning into a large scale neuromorphic architecture it is critical that the hardware required to support the learning functionality is itself scalable. This can be achieved by keeping the hardware simple and by utilizing time multiplexing to share the hardware between neurons in the simulated neural network. It is possible to efficiently integrate STDP into BrainFreeze without disrupting the functionality of the system because BrainFreeze already makes use of time-multiplexing and the required hardware additions are not individually complex. In particular, the functionality of the control component of the neuron core will need to be expanded to make decisions regarding the synapse weight updates. The control will need to determine if an update should occur as well as the degree and type of update. Some small memories will also likely be needed to store information about the learning rules that should be applied. Figure 12 depicts a potential implementation of on-line learning support hardware that is compatible with the BrainFreeze architecture. In this implementation, a shift register is used to establish the timing relationship between the pre and post synaptic spikes. If the pre-synaptic spike occurred before the post-synaptic spike then the weight of the corresponding synapse is increased. Alternatively, if the pre-synaptic spike occurs after the post-synaptic spike then the weight of the corresponding synapse is decreased. This particular implementation is included to provide a straightforward example of STDP hardware, other implementations are possible and should be explored by future work.

Beyond the changes to the control hardware on the neuron core, supporting STDP also requires additional local memory resources if a multi-temperature memory system is used. This is because the STDP hardware will need to update the weights for previous neurons before those weights can be written back to the backing store. As a result, the local buffers will need to be large enough to store the synapse weights for three or more virtual neurons. In systems that do not include STDP, the local buffers would only need to store the synapse weights for the current neuron and the weights for the next neuron that are being loaded from the backing store. In systems that do not include a multi-temperature memory system, no additional local memory is needed because all of the synapse weights for all of the simulated neurons already need to be present in the memory of the neuron core. Also, in systems that include STDP, the local buffers will need additional ports or additional banks so that a weight update can occur while other synapses are being accessed. However, these buffers will be reused by different simulated neurons due to the time-multiplexed nature of the BrainFreeze architecture so they will not greatly impact the scalability of the system. Synapse weights will require some time to be updated due to communication and access delays. In a time-multiplexed system, this update latency should be hidden since the synapses being updated will not be needed again for many clock cycles. Overall, supporting on-line learning will require additional hardware in the system but the additions should not significantly impact the scalability of the architecture.

## 12. Large System Scaling

Building upon the capabilities and configurations of BrainFreeze that have been explored so far, we can roughly estimate how well the architecture would scale for very large simulations. One of the ultimate tests of scalability for neuromorphic architectures is supporting a simulation that involves roughly 80 billion neurons. This is the approximate number of neurons contained in the human brain (Furber, 2016). In order to evaluate the scalability of BrainFreeze, we compare it to other approaches in terms of the number of standard server cabinets of equipment that would be needed to support a simulation of this size. For the purposes of this comparison we ignore the cabinets of peripheral equipment needed by the various approaches and focus only on the cabinets that contain the neuromorphic chips themselves. Many neuromorphic approaches require additional hardware to operate. Additional hardware will also likely be required to support the superconducting approach but the quantity is currently unknown. The neuromorphic chips for the BrainFreeze system studied here include the neuron core, the synapse weight memories, the post-synaptic address memories, and the routers. In addition, we include 25 cabinets that we estimate will be needed to host the roughly 1 petabyte of Cryo-DRAM that will be needed if a backing store is included in the architecture.

The scalability of superconducting neuromorphic systems like BrainFreeze is supported by several important characteristics of SCE. First, the low heat dissipation of SCE means that chips and boards can be packed much closer together than is possible in CMOS. This results in better density per unit volume even though SCE is typically less dense than CMOS in terms of area. Second, the efficiency of long distance communications in SCE means that the long wires needed to build very large systems require much less energy to operate. Third, the overall energy efficiency of SCE means that the neuron cores themselves require much less energy to operate than many CMOS alternatives. As a result of these two characteristics, much larger SCE neuromorphic systems can be built before power requirements become an obstacle. Finally, the speed of SCE means that a much higher degree of multiplexing can be employed before the run-time of the neural network simulation becomes prohibitive. This means that SCE can support more simulated neurons with less hardware. These factors combined mean that approaches like BrainFreeze are uniquely suited to scale to very large systems due to their efficient use of space, energy, and hardware.

For the superconducting system, the anticipated chip size greatly affects the amount of hardware that is required. We assume a chip size roughly equivalent to an NVidia V100 (28.5 x 28.5 mm). Chips of this size can support roughly 3 MB of a JMRAM like memory or 1,000–2,000 neurons per die. We anticipate arranging the chips on a 200 x 240 mm interposer that can accommodate 49 chips. Alternatively smaller interposers could possibly be used and connected together to build the system with a slightly higher volume overhead. Due to the limited heat generated by superconducting electronics, the boards can be placed into the racks with only 7.3 mm of space between each board.

**Figure 13:**
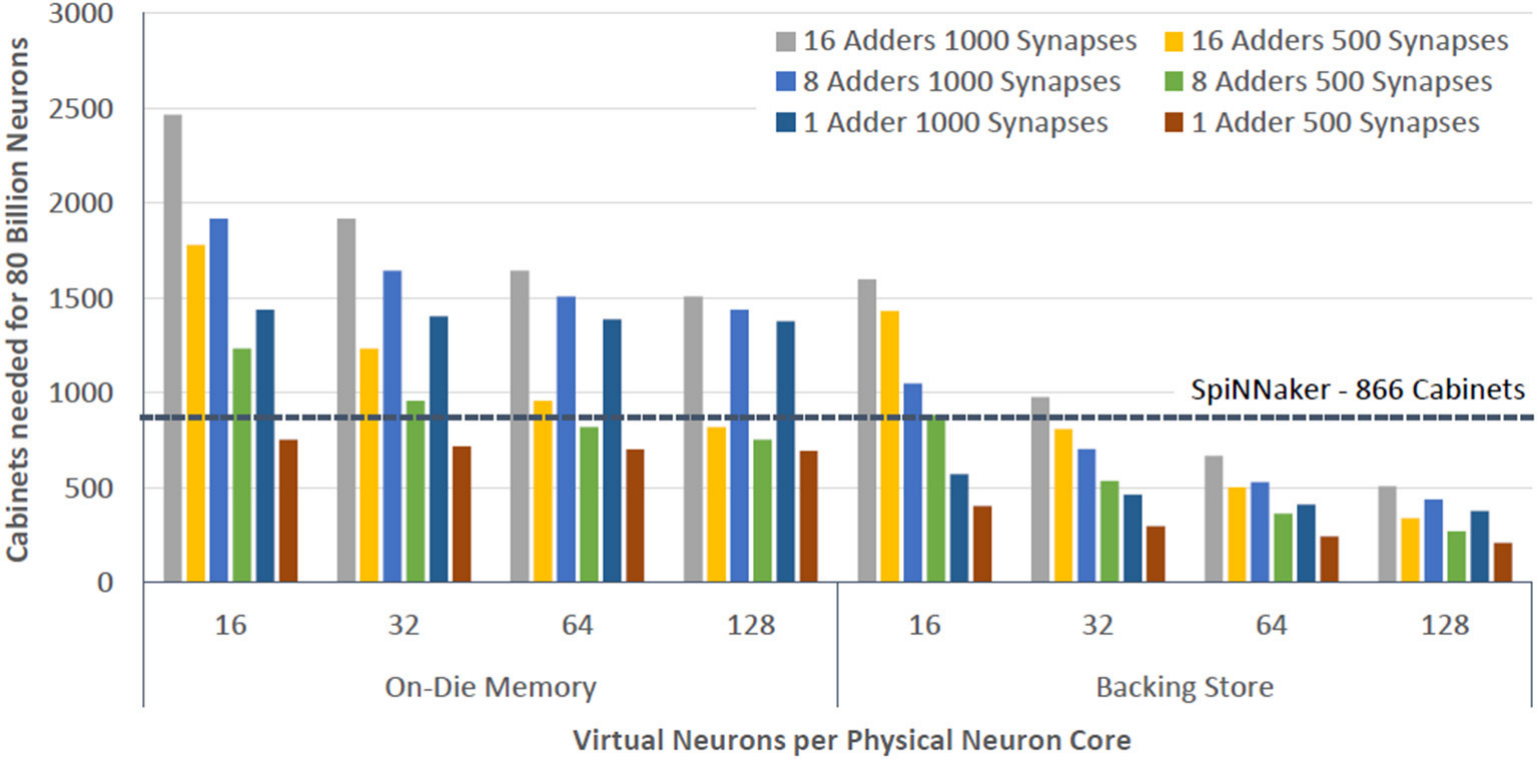
The number of standard rack sized cabinets that would be required to house an 80 billion neuron BrainFreeze system for different degrees of multiplexing. Provided that sufficient multiplexing is employed, BrainFreeze could potentially implement such a system in considerably less volume than other state-of-the-art neuromorphic approaches.

In Figure 13 we compare BrainFreeze to SpiNNaker. SpiNNaker was chosen for this comparison because it is the state-of-the-art neuromorphic approach that has been scaled to the largest number of neurons (Furber, 2016; Yang and Kim, 2020). It is worth noting that the 866 cabinets that are projected for SpiNNaker are an extrapolation of the available data. From the results in Figure 13, we can see that the number of standard server cabinets needed to house 80 billion neurons is roughly 700–2,500 if no backing store is used and 200–1,600 if the backing store is included. This illustrates the difficulty of scaling the architecture to extremely large numbers of neurons using only superconducting memories. However, with the support of a backing store it is possible to implement 80 billion neurons using roughly a quarter of the cabinets that would be required by the SpiNNaker approach. More importantly, 200 cabinets is roughly the size of a modern supercomputer. Therefore, BrainFreeze has the potential to enable extremely large neural network simulations that involve biologically relevant dynamics while requiring a volume that is suitable for a typical datacenter.

## 13. Discussion

The analysis presented in this paper shows that feasible configurations exist for the BrainFreeze architecture that would enable it to be competitive with other state-of-the-art large scale neuromorphic designs. Local superconducting memory capacity is shown to be sufficient for small scale demonstrations and for acting as a local buffer for large systems. A multi-temperature memory hierarchy combined with prefetching and the local buffers is shown to be capable of providing enough memory capacity for even large scale systems. This memory capacity and the high clock rates enabled by SCE can then be utilized to enable multiplexing to improve neuron densities per chip. Finally, the low power dissipation and subsequent low heat generation of SCE allows for more tightly packed systems ultimately resulting in a density per volume that is shown to be potentially superior to existing approaches. Taken together these results describe a system that can simultaneously provide improved performance, energy efficiency, and scalability while supporting biologically suggestive neuron models and on-line learning.

The analysis and results presented in this paper also provide motivation for future work in this area. Research is needed to continue the development of the various digital and analog components that are required to build a mixed-signal SCE neuromorphic system like BrainFreeze. For example, enabling greater configurability in the analog soma circuits could introduce new functionality to the system and expand the research that it could support. In addition, while the parameters used in this work are based on experimental demonstrations, they are still just approximations of the latency and area needed for an actual implementation of BrainFreeze. In order to validate and refine the findings of this analysis, a complete BrainFreeze core should be built in hardware. In particular, the control scheme of the BrainFreeze system needs to be carefully designed in order to preserve the important aspects of the biologically suggestive soma model despite the discretized digital communication of action potentials.

Developing large scale SCE neuromorphic systems could provide a pivotal experimental apparatus to both the machine learning and computational neuroscience communities. Enabling high performance biologically suggestive simulations could support the development of new applications and may help in the development of general AI. A large scale SCE system could be deployed as a cloud appliance thereby allowing researchers from various fields access to it. This would help to distribute the costs of developing and maintaining the system while ensuring the broadest impact of its unique collection of capabilities.

## 14. Conclusions

This work has endeavored to explain how a programmable, large scale SCE neuromorphic system could be built using a mixed-signal architecture. The feasibility of the proposed BrainFreeze architecture was supported by numerical analysis and trade studies based on measurements from experimental demonstrations. The results showed that it should be possible to build a BrainFreeze system that simultaneously provides programmability, scalability, speed, energy efficiency, biological suggestivity, and on-line learning support. Such a system could prove to be a critical resource supporting the development of novel machine learning applications, supporting computational neuroscience experiments, and perhaps one day supporting the drive to artificial general intelligence.

## Data Availability Statement

The original contributions presented in the study are included in the article/supplementary material, further inquiries can be directed to the corresponding author.

## Author Contributions

KS wrote Section 2.3, Section 4.2, and provided revisions to the other sections. PT wrote the sections of the paper not written by KS and performed the numerical analysis except for the analysis presented in Section 4.2. Both authors contributed to the article and approved the submitted version.

## Funding

This research was funded as an internal research and development effort of Northrop Grumman.

## Conflict of Interest

PT was employed by Northrop Grumman while performing this research. PT is also listed as the inventor of patent US11157804B2 - Superconducting neuromorphic core, which is related to some of the ideas discussed in this work. The remaining author declares that the research was conducted in the absence of any commercial or financial relationships that could be construed as a potential conflict of interest.

## Publisher's Note

All claims expressed in this article are solely those of the authors and do not necessarily represent those of their affiliated organizations, or those of the publisher, the editors and the reviewers. Any product that may be evaluated in this article, or claim that may be made by its manufacturer, is not guaranteed or endorsed by the publisher.

## References

[B1] Amir A. Taba B. Berg D. Melano T. McKinstry J. Di Nolfo C.. (2017). “A low power, fully event-based gesture recognition system,” in Proceedings of the IEEE Conference on Computer Vision and Pattern Recognition (Honolulu, HI: IEEE), 7243–7252.

[B2] Amodei D. Ananthanarayanan S. Anubhai R. Bai J. Battenberg E. Case C.. (2016). “Deep speech 2: End-to-end speech recognition in english and mandarin,” in International Conference on Machine Learning (New York, NY: PMLR), 173–182.

[B3] Amodei D. Hernandez D. Sastry G. Clark J. Brockman G. Sutskever I. (2018). Ai and Compute. Available online: https://blog.openai.com/aiand-compute.

[B4] Aradhya S. V. Rowlands G. E. Oh J. Ralph D. C. Buhrman R. A. (2016). Nanosecond-timescale low energy switching of in-plane magnetic tunnel junctions through dynamic oersted-field-assisted spin hall effect. Nano Lett. 16, 5987–5992. 10.1021/acs.nanolett.6b01443 27327619

[B5] Arthur J. V. Boahen K. A. (2011). Silicon-neuron design: a dynamical systems approach. IEEE Trans. Circ. Syst. I. 58, 1034–1043. 10.1109/TCSI.2010.2089556 21617741 PMC3100558

[B6] Benjamin B. V. Gao P. McQuinn E. Choudhary S. Chandrasekaran A. R. Bussat J.. (2014). Neurogrid: a mixed-analog-digital multichip system for large-scale neural simulations. Proc. IEEE 102, 699–716. 10.1109/JPROC.2014.231356527295638

[B7] Boahen K.. (2000). Point-to-point connectivity between neuromorphic chips using address events. IEEE Tran. Circ. Sys. II 47:416. 10.1109/82.84211027295638

[B8] Borkar S.. (2011). “The exascale challenge. keynote presentation at PACT,” in IEEE International Conference on Parallel Architectures and Compilation Techniques (PACT) (Galveston, TX).

[B9] Buckley S. Chiles J. McCaughan A. N. Moody G. Silverman K. L. Stevens M. J.. (2017). All-silicon light-emitting diodes waveguide-integrated with superconducting single-photon detectors. Appl. Phys. Lett. 111, 141101. 10.1063/1.4994692 36452265 PMC9706689

[B10] Buckley S. McCaughan A. N. Chiles J. Mirin R. P. Nam S. W. Shainline J. M.. (2018). “Design of superconducting optoelectronic networks for neuromorphic computing,” in 2018 IEEE International Conference on Rebooting Computing (ICRC) (McLean, VA: IEEE), 1–7.

[B11] Buckley S. M. Chiles J. McCaughan A. N. Mirin R. P. Nam S. W. Shainline J. M. (2017). “Photonic interconnect with superconducting electronics for large-scale neuromorphic computing (invited paper),” in 2017 IEEE Photonics Society Summer Topical Meeting Series (SUM) (San Juan, PR). 51–52.

[B12] Burnett R. Clarke R. Lee T. Hearne H. Vogel J. Herr Q.. (2018). “Demonstration of superconducting memory for an rql cpu,” in Proceedings of the International Symposium on Memory Systems, MEMSYS '18 (New York, NY: ACM), 321–323.

[B13] Cassidy A. S. Georgiou J. Andreou A. G. (2013a). Design of silicon brains in the nano-cmos era: spiking neurons, learning synapses and neural architecture optimization. Neural Netw. 45:4–26. 10.1016/j.neunet.2013.05.011 23886551

[B14] Cassidy A. S. Merolla P. Arthur J. V. Esser S. K. Jackson B. Alvarez-Icaza R.. (2013b). “Cognitive computing building block: a versatile and efficient digital neuron model for neurosynaptic cores,” in The 2013 International Joint Conference on Neural Networks (IJCNN) (Dallas, TX: IEEE), 1–10.

[B15] Chiles J. Buckley S. M. Nam S. W. Mirin R. P. Shainline J. M. (2018). Design, fabrication, and metrology of 10 100 multi-planar integrated photonic routing manifolds for neural networks. APL Photonics 3, 106101. 10.1063/1.503964124313031

[B16] Chollet F.. (2017). “Xception: deep learning with depthwise separable convolutions,” in Proceedings of the IEEE Conference on Computer Vision and Pattern Recognition (Honolulu, HI: IEEE), 1251–1258.

[B17] Crotty P. Schult D. Segall K. (2010). Josephson junction simulation of neurons. Phys. Rev. E 82:011914. 10.1103/PhysRevE.82.011914 20866655

[B18] Davies M. Srinivasa N. Lin T. Chinya G. Cao Y. Choday S. H.. (2018). Loihi: A neuromorphic manycore processor with on-chip learning. IEEE Micro 38, 82–99. 10.1109/MM.2018.11213035927295638

[B19] Dayton I. Baker H. Loving M. Ambrose T. Siwak N. Keebaugh S.. (2018). “Demonstration of josephson magnetic random access memory (jmram),” in Applied Superconductivity Conference (Seattle, WA).

[B20] Dayton I. M. Sage T. Gingrich E. C. Loving M. G. Ambrose T. F. Siwak N. P.. (2018). Experimental demonstration of a josephson magnetic memory cell with a programmable π-junction. IEEE Magn. Lett. 9, 1–5. 10.1109/LMAG.2018.280182027295638

[B21] Ebong I. E. Mazumder P. (2012). Cmos and memristor-based neural network design for position detection. Proc. IEEE 100, 2050–2060. 10.1109/JPROC.2011.217308927295638

[B22] Furber S.. (2016). Large-scale neuromorphic computing systems. J. Neural Eng. 13, 051001. 10.1088/1741-2560/13/5/051001 27529195

[B23] Furber S. B. Galluppi F. Temple S. Plana L. A. (2014). The spinnaker project. Proc. IEEE 102, 652–665. 10.1109/JPROC.2014.230463827295638

[B24] Furber S. B. Lester D. R. Plana L. A. Garside J. D. Painkras E. Temple S.. (2013). Overview of the spinnaker system architecture. IEEE Trans. Comput. 62, 2454–2467. 10.1109/TC.2012.14227295638

[B25] He K. Zhang X. Ren S. Sun J. (2016). “Deep residual learning for image recognition,” in Proceedings of the IEEE Conference on Computer Vision and Pattern Recognition (Las Vegas, NV: IEEE), 770–778.

[B26] Hearne H. Vogel J. Clarke R. Burnett R. Lee T. Herr A.. (2018). “Rql encoded 8x16 register file for 16-bit cpu,” in Applied Superconductivity Conference (Seattle, WA).

[B27] Herr Q. P. Herr A. Y. Oberg O. T. Ioannidis A. G. (2011). Ultra-low-power superconductor logic. J. Appl. Phys. 109, 103903. 10.1063/1.358584924313031

[B28] Hidaka M. Akers L. A. (1991). An artificial neural cell implemented with superconducting circuits. Supercond. Sci. Technol. 4, 654–657. 10.1088/0953-2048/4/11/027

[B29] Hinton G. E. Srivastava N. Krizhevsky A. Sutskever I. Salakhutdinov R. R. (2012). Improving neural networks by preventing co-adaptation of feature detectors. arXiv preprint arXiv:1207.0580.

[B30] Hodgkin A. Huxley A. (1990). A quantitative description of membrane current and its application to conduction and excitation in nerve (reprinted from journal of physiology, vol 117, pg 500-544, 1952). Bull. Math. Biol. 52, 25–71. 10.1016/S0092-8240(05)80004-7 2185861

[B31] Inoue K. Takeuchi N. Ehara K. Yamanashi Y. Yoshikawa N. (2013). Simulation and experimental demonstration of logic circuits using an ultra-low-power adiabatic quantum-flux-parametron. IEEE Trans. Appl. Supercond. 23, 1301105–1301105. 10.1109/TASC.2012.223613327295638

[B32] Inoue K. Takeuchi N. Narama T. Yamanashi Y. Yoshikawa N. (2015). Design and demonstration of adiabatic quantum-flux-parametron logic circuits with superconductor magnetic shields. Supercond. Sci. Technol. 28, 045020. 10.1088/0953-2048/28/4/045020

[B33] Izhikevich E. M.. (2004). Which model to use for cortical spiking neurons? IEEE Trans. Neural Netw. 15, 1063–1070. 10.1109/TNN.2004.832719 15484883

[B34] Kelly T. Fernandez P. Vogelsang T. McKee S. A. Gopalakrishnan L. Magee S.. (2019). Some like it cold: Initial testing results for cryogenic computing components. J. Phys. 1182:012004. 10.1088/1742-6596/1182/1/012004

[B35] Kirichenko A. F. Sarwana S. Vernik I. V. (2012). “Ersfq-zero static power dissipation single flux quantum logic,” in Government Microcircuit Applications and Critical Technology Conference (GOMACTech-12) (Las Vegas, NV), 319–322.

[B36] Krizhevsky A. Sutskever I. Hinton G. E. (2017). Imagenet classification with deep convolutional neural networks. Commun. ACM. 60, 84–90. 10.1145/3065386

[B37] Lin C.-K. Wild A. Chinya G. N. Lin T.-H. Davies M. Wang H. (2018). “Mapping spiking neural networks onto a manycore neuromorphic architecture,” in Proceedings of the 39th ACM SIGPLAN Conference on Programming Language Design and Implementation, PLDI 2018 (New York, NY: ACM), 78–89.

[B38] Mahowald M.. (1992). VLSI Analogs of Neuronal Visual Processing: A Synthesis of Form and Function. Pasadena, CA: California Institute of Technology, (Unpublished). Available online at: https://resolver.caltech.edu/CaltechCSTR:1992.cs-tr-92-15

[B39] Makhlooghpour A. Soleimani H. Ahmadi A. Zwolinski M. Saif M. (2016). “High accuracy implementation of adaptive exponential integrated and fire neuron model,” in 2016 International Joint Conference on Neural Networks (IJCNN) (Vancouver, BC: IEEE), 192–197.

[B40] Manheimer M. A.. (2015). Cryogenic computing complexity program: Phase 1 introduction. IEEE Trans. Appl. Supercond. 25, 1–4. 10.1109/TASC.2015.239986632863691

[B41] Meier K.. (2015). “A mixed-signal universal neuromorphic computing system,” in 2015 IEEE International Electron Devices Meeting (IEDM) (Washington, DC), 4.6.1–4.6.4.

[B42] Merolla P. A. Arthur J. V. Alvarez-Icaza R. Cassidy A. S. Sawada J. Akopyan F.. (2014). A million spiking-neuron integrated circuit with a scalable communication network and interface. Science 345, 668–673. 10.1126/science.1254642 25104385

[B43] Mizugaki Y. Nakajima K. Sawada Y. Yamashita T. (1993). Superconducting neural circuits using fluxon pulses. Appl. Phys. Lett. 62, 762–764. 10.1063/1.10857124313031

[B44] Mizugaki Y. Nakajima K. Sawada Y. Yamashita T. (1994). Implementation of new superconducting neural circuits using coupled squids. IEEE Trans. Appl. Supercond. 4, 1–8. 10.1109/77.273058

[B45] Mnih V. Kavukcuoglu K. Silver D. Graves A. Antonoglou I. Wierstra D.. (2013). Playing atari with deep reinforcement learning. arXiv preprint arXiv:1312.5602.

[B46] Nagumo J. Arimoto S. Yoshizawa S. (1962). Impulses and physiological states in models of nerve membrane. Proc. Inst. Radio Engrs 50, 2061–2070. 10.1109/JRPROC.1962.28823519431309

[B47] Narama T. Yamanashi Y. Takeuchi N. Ortlepp T. Yoshikawa N. (2015). “Demonstration of 10k gate-scale adiabatic-quantum-flux-parametron circuits,” in 2015 15th International Superconductive Electronics Conference (ISEC) (Nagoya), 1–3.

[B48] Park J. Yu T. Joshi S. Maier C. Cauwenberghs G. (2017). Hierarchical address event routing for reconfigurable large-scale neuromorphic systems. IEEE Trans. Neural Netw. Learn. Syst. 28:2408. 10.1109/TNNLS.2016.2572164 27483491

[B49] Qian G. Cahay M. Kothari R. (1995). A new superconducting neural cell. Superlattices Microstruct. 18, 259. 10.1006/spmi.1995.1110

[B50] Rast A. D. Shufan Y.ang Khan M. Furber S. B. (2008). “Virtual synaptic interconnect using an asynchronous network-on-chip,” in 2008 IEEE International Joint Conference on Neural Networks (IEEE World Congress on Computational Intelligence) (Hong Kong: IEEE), 2727–2734.

[B51] Rippert E. Lomatch S. (1997). A multilayered superconducting neural network implementation. IEEE Trans. Appil. Supercond. 7, 3442–3445. 10.1109/77.62212627295638

[B52] Rose R. Hindmarsh J. (1989). The assembly of ionic currents in a thalamic neuron i. the three-dimensional model. Proc. R. Soc. Lond. B Biol. Sci. 237, 267–288. 10.1098/rspb.1989.0049 2571154

[B53] Schemmel J. Briiderle D. Griibl A. Hock M. Meier K. Millner S. (2010). “A wafer-scale neuromorphic hardware system for large-scale neural modeling,” in Proceedings of 2010 IEEE International Symposium on Circuits and Systems (Paris), 1947–1950.

[B54] Schneider M. Segall K. (2020). Fan-out and fan-in properties of superconducting neuromorphic circuits. J. Appl. Phys. 128, 214903. 10.1063/5.002516834819835

[B55] Schneider M. L. Donnelly C. A. Russek S. E. (2018a). Tutorial: high-speed low-power neuromorphic systems based on magnetic josephson junctions. J. Appl. Phys. 124, 161102. 10.1063/1.5042425

[B56] Schneider M. L. Donnelly C. A. Russek S. E. Baek B. Pufall M. R. Hopkins P. F.. (2018b). Ultralow power artificial synapses using nanotextured magnetic josephson junctions. Sci. Adv. 4, e1701329. 10.1126/sciadv.1701329 29387787 PMC5786439

[B57] Sebastian A. Le Gallo M. Burr G. W. Kim S. BrightSky M. Eleftheriou E. (2018). Tutorial: Brain-inspired computing using phase-change memory devices. J. Appl. Phys. 124, 111101. 10.1063/1.504241324313031

[B58] Shainline J. M.. (2020). Fluxonic processing of photonic synapse events. IEEE J. Select. Top. Quantum Electron. 26, 1–15. 10.1109/JSTQE.2019.292747327295638

[B59] Shainline J. M. Buckley S. M. McCaughan A. N. Chiles J. T. Salim A. J. Castellanos-Beltran M.. (2019). Superconducting optoelectronic loop neurons. J. Appl. Phys. 126, 044902. 10.1063/1.509640324313031

[B60] Shainline J. M. Buckley S. M. Mirin R. P. Nam S. W. (2016). “Neuromorphic computing with integrated photonics and superconductors,” in 2016 IEEE International Conference on Rebooting Computing (ICRC) (San Diego, CA), 1–8.

[B61] Silver D. Hubert T. Schrittwieser J. Antonoglou I. Lai M. Guez A.. (2017a). Mastering chess and shogi by self-play with a general reinforcement learning algorithm. arXiv preprint arXiv:1712.01815. 10.1126/science.aar6404 30523106

[B62] Silver D. Schrittwieser J. Simonyan K. Antonoglou I. Huang A. Guez A.. (2017b). Mastering the game of go without human knowledge. Nature 550, 354–359. 10.1038/nature24270 29052630

[B63] Simonyan K. Zisserman A. (2014). Very deep convolutional networks for large-scale image recognition. arXiv preprint arXiv:1409.1556.

[B64] Sompolinsky H.. (2014). Computational neuroscience: beyond the local circuit. Curr. Opin. Neurobiol. 25, xiii–xviii. 10.1016/j.conb.2014.02.002 24602868

[B65] Soudry D. Di Castro D. Gal A. Kolodny A. Kvatinsky S. (2015). Memristor-based multilayer neural networks with online gradient descent training. IEEE Trans. Neural Netw. Learn. Syst. 26, 2408–2421. 10.1109/TNNLS.2014.2383395 25594981

[B66] Stromatias E. Neil D. Galluppi F. Pfeiffer M. Liu S.-C. Furber S. (2015). “Scalable energy-efficient, low-latency implementations of trained spiking deep belief networks on spinnaker,” in 2015 International Joint Conference on Neural Networks (IJCNN) (Killarney: IEEE), 1–8.

[B67] Sutskever I. Vinyals O. Le Q. V. (2014). Sequence to sequence learning with neural networks. arXiv preprint arXiv:1409.3215.

[B68] Szegedy C. Liu W. Jia Y. Sermanet P. Reed S. Anguelov D.. (2015). “Going deeper with convolutions,” in Proceedings of the IEEE Conference on Computer Vision and Pattern Recognition (Boston, MA: IEEE), 1–9.

[B69] Tannu S. S. Carmean D. M. Qureshi M. K. (2017). “Cryogenic-dram based memory system for scalable quantum computers: a feasibility study,” in Proceedings of the International Symposium on Memory Systems, MEMSYS '17 (New York, NY: ACM), 189–195.

[B70] Vesely M. Tschirhart P. Clarke R. Huertas-Morales Y. Lateef M. Rahman S.. (2018). “An 8-bit and 16-bit alu for superonducting reciprocal quantum logic (rql) cpus,” in Applied Superconductivity Conference (Seattle, WA).

[B71] Vinyals O. Babuschkin I. Czarnecki W. M. Mathieu M. Dudzik A. Chung J.. (2019). Grandmaster level in starcraft ii using multi-agent reinforcement learning. Nature 575, 350–354. 10.1038/s41586-019-1724-z 31666705

[B72] Wang F. Vogelsang T. Haukness B. Magee S. C. (2018). “Dram retention at cryogenic temperatures,” in 2018 IEEE International Memory Workshop (IMW) (Kyoto: IEEE), 1–4.

[B73] Ware F. Gopalakrishnan L. Linstadt E. McKee S. A. Vogelsang T. Wright K. L.. (2017). “Do superconducting processors really need cryogenic memories?: the case for cold dram,” in Proceedings of the International Symposium on Memory Systems, MEMSYS '17 (New York, NY: ACM), 183–188.

[B74] Wu Y. Schuster M. Chen Z. Le Q. V. Norouzi M. Macherey W.. (2016). Google's neural machine translation system: Bridging the gap between human and machine translation. arXiv preprint arXiv:1609.08144.

[B75] Yang Y. S. Kim Y. (2020). “Recent trend of neuromorphic computing hardware: Intel's neuromorphic system perspective,” in 2020 International SoC Design Conference (ISOCC) (Yeosu: IEEE), 218–219.

[B76] Ye L. Gopman D. Rehm L. Backes D. Wolf G. Ohki T.. (2014). Spin-transfer switching of orthogonal spin-valve devices at cryogenic temperatures. J. Appl. Phys. 115, 17C725. 10.1063/1.486546424313031

[B77] Young A. R. Dean M. E. Plank J. S. Rose G. S. (2019). A review of spiking neuromorphic hardware communication systems. IEEE Access. 7, 135606–135620. 10.1109/ACCESS.2019.294177226422422

[B78] Zeiler M. D. Fergus R. (2014). “Visualizing and understanding convolutional networks,” in European Conference on Computer Vision (Zurich: Springer), 818–833.

[B79] Zoph B. Le Q. V. (2016). Neural architecture search with reinforcement learning. arXiv preprint arXiv:1611.01578.

